# Levels of Zinc, Iron, and Copper in the Aqueous Humor of Patients with Primary Glaucoma

**DOI:** 10.3390/biom15070962

**Published:** 2025-07-04

**Authors:** Yangjiani Li, Zhe Liu, Zhidong Li, Yingting Zhu, Shuxin Liang, Hongtao Liu, Jingfei Xue, Jicheng Lin, Ye Deng, Caibin Deng, Simei Zeng, Yehong Zhuo, Yiqing Li

**Affiliations:** 1 State Key Laboratory of Ophthalmology, Zhongshan Ophthalmic Center, Sun Yat-Sen University, Guangdong Provincial Key Laboratory of Ophthalmology and Visual Science, Guangzhou 510060, China; liyjn@mail2.sysu.edu.cn (Y.L.); liuzh253@mail2.sysu.edu.cn (Z.L.); lizhd7@mail.sysu.edu.cn (Z.L.); zhuyt35@mail.sysu.edu.cn (Y.Z.); rmhaian@ucl.ac.uk (S.L.); xuejf3@mail3.sysu.edu.cn (J.X.); linjch29@mail2.sysu.edu.cn (J.L.); ddendxx@126.com (Y.D.); dengcb@mail.sysu.edu.cn (C.D.); zengsimei023@163.com (S.Z.); 2 Guangdong Provincial Clinical Research Center for Ocular Diseases, Guangzhou 510060, China; 3 Instrumental Analysis and Research Center, Sun Yat-Sen University, Guangzhou 510275, China; liuht@mail.sysu.edu.cn

**Keywords:** glaucoma, aqueous humor, zinc, iron, copper

## Abstract

Background: This case–control study evaluated the concentrations of zinc (Zn), iron (Fe), and copper (Cu) in the aqueous humor (AH) of patients with primary glaucoma, and their relationships with clinical factors. Methods: This study enrolled 100 patients with primary glaucoma and categorized them into subtypes: acute angle-closure crisis (AACC), primary angle-closure glaucoma (PACG), and primary open-angle glaucoma (POAG). A total of 67 patients with senile cataract were enrolled as controls. Their AH samples and clinical information were obtained. Results: In primary glaucoma, Zn, Fe, and Cu concentrations increased, especially in AACC group; Zn, Fe, and Cu were positively correlated mutually; and decreased Zn/Fe and increased Fe/Cu were observed. The number of quadrants with closed anterior chamber angle on gonioscopy was positively associated with Fe and Cu levels in AACC and with Zn and Cu levels in PACG. In POAG, we found negative associations between Zn and the number of quadrants with retinal nerve fiber layer thinning on optical coherence tomography, Fe and age, and Cu and the cup-to-disc ratio. Trace metals showed high efficiency in discriminating primary glaucoma from controls. Conclusions: Zn, Fe, and Cu concentrations in patients with primary glaucoma increased and were associated with clinical factors, acting as potential biomarkers.

## 1. Introduction

Glaucoma is the most common cause of irreversible blindness globally [1]. The estimated number of glaucoma patients (aged 40–80 years) worldwide will increase to 111.8 million by 2040 [2]. Glaucoma is characterized by degeneration of the retinal ganglion cells (RGCs) and their axons. Common pathological changes involve dysfunction of the trabecular meshwork (TM) [3], hemodynamic changes [4], immune dysregulation [5], oxidative stress [6], and glial dysfunction [7]. Risk factors include elevated intraocular pressure (IOP), aging, race, a family history of glaucoma, and specific medications [8]. Primary glaucoma is the most common form of glaucoma. It can be classified into primary angle-closure glaucoma (PACG) and primary open-angle glaucoma (POAG). The acute angle-closure crisis (AACC), manifested as an abrupt elevation of IOP, eye pain, and blurred vision, is an acute angle closure attack and can develop into PACG [9].

Trace metals, constituting less than 0.01% of human body weight, are critical for ocular homeostasis. The dysregulation of trace metals has been implicated in the pathogenesis of various eye diseases, including glaucoma. This study focused on three essential trace metals: zinc (Zn), iron (Fe), and copper (Cu).

Zn is a crucial component of metalloenzymes. Carbonic anhydrase, a zinc-dependent metalloenzyme regulating physiological aqueous humor balance, is a treatment target in glaucoma [10]. Matrix metalloproteinases (MMPs), Zn-dependent endopeptidases, participate in the degradation of the extracellular matrix (ECM) proteins and the processing of several bioactive protein molecules. Zn has been proven to regulate the ECM of TM in dexamethasone-induced glaucoma [3].

Fe modulates numerous enzyme and protein activities [11]. Both Fe and Zn regulate mitochondrial function in glaucoma [12]. Fe is a target for both nitrogen monoxide and carbon monoxide in glaucoma [13]. Fe can induce oxidative stress and ferroptosis, potentially contributing to lipid peroxidation, DNA damage, biomolecules degradation, and RGC death. Additionally, TM cells’ senescence and ECM remodeling are influenced by Fe and ferroptosis, leading to elevation of IOP [14].

Cu is related to retinal physiology and functions. Cu participates in Fenton’s reaction, generating highly reactive hydroxyl radicals [15]. Excessive Cu can trigger retinal inflammation, cell membranes damage, and mitochondrial dysfunction. Interactions between Cu and nitric oxide synthase, N-methyl-D-aspartic acid (NMDA), and α-amino-3-hydroxy-5-methyl-4-isoxazolepropionic acid (AMPA) receptors may contribute to retinal neuron death [16].

While serum levels of Zn, Fe, and Cu in glaucoma have been studied, the blood-aqueous barrier limits the association between serum and intraocular conditions. Compared to serum, aqueous humor (AH) offers a more sensitive reflection of ocular diseases. With relatively simple sampling and minimal clinical risk, AH-based analysis is well-established in proteomics [17], metabolomics [18], and genomics [19]. This indicates the feasibility of AH components detection and the potential of AH components as a good source for biomarkers of ocular diseases.

The association between Zn, Fe, and Cu in AH and glaucoma has been reported [20,21,22,23], but findings are inconsistent. Different studies report higher or unchanged Zn, Fe, and Cu levels in the AH of patients with POAG, potentially due to methodological or population differences. Some researchers note mutual associations between trace metals and the links between trace metal concentrations and demographic factors like age and sex. Compared with POAG, the trace metal levels in AH of PACG and AACC patients are less documented. Therefore, further evidence is needed to clarify the association between trace metals and primary glaucoma.

This study quantified and compared Zn, Fe, and Cu concentrations in the AH of patients diagnosed as primary glaucoma (AACC, PACG, and POAG) and senile cataract (controls) using the flow injection inductively coupled plasma mass spectrometry (FI-ICP-MS). We examined mutual correlations between trace metals, investigated their associations with clinical factors, and evaluated their potential as biomarkers. Our findings aim to enhance understanding of the roles played by trace metal in primary glaucoma.

## 2. Materials and Methods

### 2.1. Study Design

This study was conducted in accordance with the Declaration of Helsinki and was approved by the Institutional Review Board of Zhongshan Ophthalmic Center, Sun Yat-sen University (registration number: 2020KYPJ120). This study was conducted at the Zhongshan Ophthalmic Center from 20 August 2020 to 20 October 2022. Written informed consent was obtained from all participants. This study was registered at ClinicalTrials.gov (trial registration number: NCT04515030; registration date: 10 August 2020; registration site: Zhongshan Ophthalmic Center, Guangzhou, China; URL: https://clinicaltrials.gov/study/NCT04515030 accessed on 10 May 2025).

### 2.2. Participants

All patients were diagnosed based on the guidelines [24,25,26]. Patients with primary glaucoma were categorized into the AACC, PACG, and POAG subtypes. Patients with senile cataract were enrolled as controls. Only one eye per patient was included. If both eyes underwent surgery, the first operated eye was selected. Exclusion criteria included the following: (1) a history of ocular trauma, (2) previous eye surgeries (including laser treatment) in the last 6 months, (3) secondary or developmental factors that may increase IOP (e.g., neovascularization, uveitis, and trauma), (4) other ocular or systemic diseases that may affect the concentration of trace metals (e.g., gastrointestinal malabsorption, hypothyroidism, and retinoblastoma), (5) history of medication or supplements that may affect the levels of trace metal (e.g., folic acid antagonists), and (6) pregnancy (current or planned) or lactation.

### 2.3. Clinical Variables

The demographic and clinical data of all patients were collected preoperatively. Visual acuity (VA) (5m Standard Logarithmic Visual Acuity chart [27], Yuehua Inc., Shantou, China), IOP (Goldmann tonometer, Liuliu Inc., Suzhou, China), and cup-to-disc ratio (CDR) were obtained. The VA was transformed into the logarithm of minimum angle of resolution (logMAR) for statistical analysis. The IOP was measured by an experienced ophthalmologist using a Goldmann tonometer on the day of surgery in the inpatient ward of the Glaucoma Department. For each eye, IOP was the average value of three eligible measurements. The axial length (AL) was measured using an A-scan ultrasound biometer (CineScan A/B-scan, Quantel Medical, Cournon-d’Auvergne, France). The retinal nerve fiber layer (RNFL) thickness was obtained by optical coherence tomography (OCT) (Spectralis SD-OCT, Heidelberg Engineering, Heidelberg, Germany) and was quantified using the number of quadrants with RNFL thinning on OCT. Patients with a narrow anterior chamber angle (ACA) underwent gonioscopy and ultrasound biomicroscopy (UBM) (full scan UBM, Suoer, Tianjin, China), by which anterior chamber depth (ACD) was measured. Angle closure was quantified using the number of quadrants with closed ACA on gonioscopy or UBM, respectively. The mean deviation (MD) and pattern standard deviation (PSD) were obtained using visual field (VF) tests (Humphrey Field Analyzer, Zeiss, Jena, Germany) conducted on patients with chronic glaucoma (PACG and POAG subtypes). Age, logMAR, IOP, AL, and duration of disease were obtained for patients with senile cataracts. AH samples were obtained from all patients.

### 2.4. Sample Preparation and Quantitative Analysis of Trace Metals

Enrolled patients underwent surgeries, including phacoemulsification with intraocular lens implantation, trabeculectomy, glaucoma implant surgery, and combined cataract–glaucoma surgery. Despite the types of surgery, AH samples were collected via anterior chamber paracentesis using a 1 mL single-use syringe (KDL, Shanghai, China) and a 28 G needle (KDL, Shanghai, China) before any other invasive intraocular procedure, ensuring that the samples were unaffected by surgery. A senior ophthalmologist performed the AH sampling and surgery.

AH samples were aseptically transferred into Eppendorf tubes (Company Axygen, Corning, NY, USA, cat# MCT-150-C-S) and immediately stored at −80 °C. A volume of 50 μL of AH was mixed with 50 μL of nitric acid (HNO_3_)-based germanium (Ge), scandium (Sc), and indium (In) internal standard solution to reach final concentrations of 40 μg/L Ge, 40 μg/L Sc, 40 μg/L In, and 2% HNO_3_. Then, it was mixed with a 100 μL solution containing 1% Triton X-100, 2% methanol, and 2% HNO_3_. A series of identical standard solutions and sample blanks were prepared. An inductively coupled plasma mass spectrometer (ICP-MS) (iCap Q, Thermo Fisher, Waltham, MA, USA) and an automated flow injection system (prepFAST M5, Elemental Scientific, USA) were used for the quantitative analysis of trace metals in the AH. Quality control was applied. A low AH volume can lead to inaccurate quantitative detection of trace metals; thus, patients with low-quality trace metal detection were excluded.

### 2.5. Statistical and Data Analysis

R 3.6.1 (R Foundation for Statistical Computing, Austria) was used for the analysis. Because the trace metal concentrations showed a log-normal distribution [28,29], we used the natural logarithmic (ln) transformation for Zn, Fe, and Cu (ln[Zn], ln[Fe], and ln[Cu], respectively) for subsequent statistical analysis. Normal distribution was verified using the Shapiro–Wilk test. An unpaired two-tailed Student’s *t*-test and one-way ANOVA followed by Dunnett-T3 or Scheffé post hoc tests, Mann–Whitney U test, Kruskal–Wallis test, and median test were performed for comparison (details are presented in footnotes). Spearman’s correlation analysis was also performed. Clinical variables with significant correlations with trace metal concentrations were included in a backward stepwise multivariate linear regression analysis based on the Bayesian information criterion. A receiver operating characteristic (ROC) curve was plotted, and logistic regression analysis was performed to evaluate the discriminative potential of the trace metals. The area under the ROC curve (AUC) and odds ratios (ORs) were also calculated. A *p*-value less than 0.05 was considered statistically significant.

## 3. Results

### 3.1. Participant Characteristics

A total of 193 patients were recruited, of whom 26 were excluded because of secondary or developmental factors that may increase IOP (5 patients), previous eye surgeries (6 patients), and low volume of AH (15 patients). Finally, 100 patients with primary glaucoma (22, 42, and 36 in the AACC, PACG, and POAG subtypes, respectively) and 67 patients with senile cataract were enrolled. Table 1 summarizes the patients’ basic characteristics. Table 2 shows the distribution of Zn, Fe, and Cu concentrations and their natural logarithmic transformations. The patients’ sex and age differed among the subtypes. However, the partial correlation analysis (Tables S1 and S2) indicated no significant relationship between age or sex and the Zn, Fe, and Cu concentrations in any group. The types of IOP-lowering drugs were summarized in Table S3. The AACC group used less travoprost and more mannitol than the POAG group; the PACG group used less pilocarpine, bimatoprost, and travoprost, and more mannitol than the POAG group. However, the number of IOP-lowering drugs did not differ significantly between glaucoma subgroups.

### 3.2. Changes in Trace Metal Concentrations in the AH

**Figure 1:**
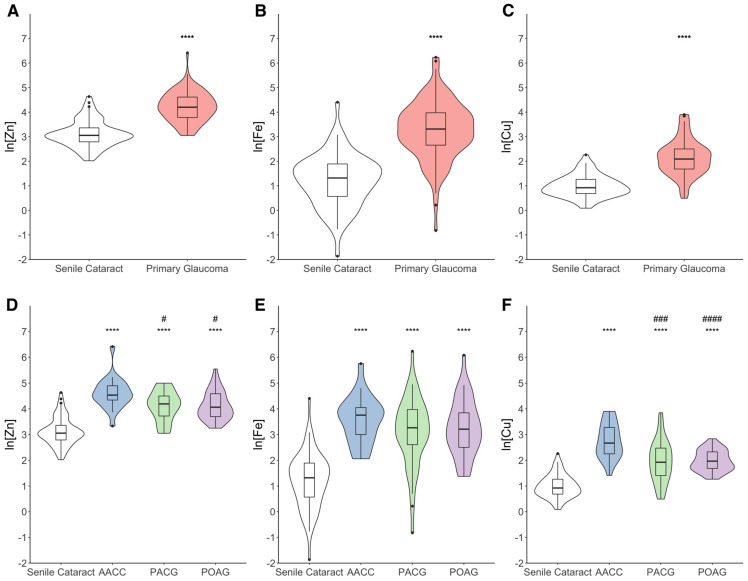
The trace metal concentrations in the aqueous humor of primary glaucoma patients and controls. (**A**–**C**) Levels of Zn, Fe, and Cu were higher in the AH of primary glaucoma patients when compared with those in the AH of controls. The natural logarithmic conversion was performed. Statistical analysis by Student’s *t*-test. **** *p <* 0.001. (**D**–**F**) Zn, Fe, and Cu levels in the AH were higher in all three subtypes of primary glaucoma patients compared with those in the AH of controls. The AACC group shows greater change in trace metal concentrations than the PACG and POAG groups. The natural logarithmic conversion was performed. Statistical analysis by one-way ANOVA followed by Scheffé post hoc test for Zn and Fe, and Dunnett T3 post hoc test for Cu. **** *p* < 0.001 compared with the control group; #### *p* < 0.001, ### *p* < 0.005, # *p* < 0.05 compared with the AACC group. Abbr.: AACC = acute angle-closure crisis; PACG = primary angle-closure glaucoma; POAG = primary open-angle glaucoma; ln = natural logarithmic transformation; Zn = zinc; Fe = iron; Cu = copper.

We compared Zn, Fe, and Cu concentrations in the AH of patients with primary glaucoma and those with senile cataract (control). As shown in Figure 1A–C, the levels of Zn (ln[Zn]: 4.22 ± 0.06, +35.46%), Fe (ln[Fe]: 3.26 ± 0.12, +169.36%), and Cu (ln[Cu]: 2.15 ± 0.07, +117.33%) in the primary glaucoma group were significantly higher than that in the senile cataract group (*p* < 0.001). In particular, the levels of Zn, Fe, and Cu in the AH were higher in all three glaucoma subtypes (*p* < 0.001) (Figure 1D–F). The AACC group showed the highest concentration of Zn (ln[Zn]: 4.60 ± 0.13), Fe (ln[Fe]: 3.58 ± 0.19), and Cu (ln[Cu]: 2.76 ± 0.15). The Zn and Cu levels in the AACC group were significantly higher than those in the PACG and POAG groups (*p* < 0.05).

An elevated IOP is a major risk factor for glaucoma. Hence, all patients were divided according to their IOP level. Zn (ln[Zn]: 4.19 ± 0.09, +15.92%), Fe (ln[Fe]: 3.45 ± 0.19, +65.42%), and Cu (ln[Cu]: 2.21 ± 0.11, +50.34%) concentrations were higher in the group with IOP larger than 21 mmHg (*p* < 0.001) (Figure S1). However, after adjusting for glaucoma subtypes as a confounding factor, the effect of IOP was not significant (Table S4), suggesting an additional influence of glaucoma subtypes.

### 3.3. Relationship Between Trace Metal Concentrations in the AH

**Figure 2:**
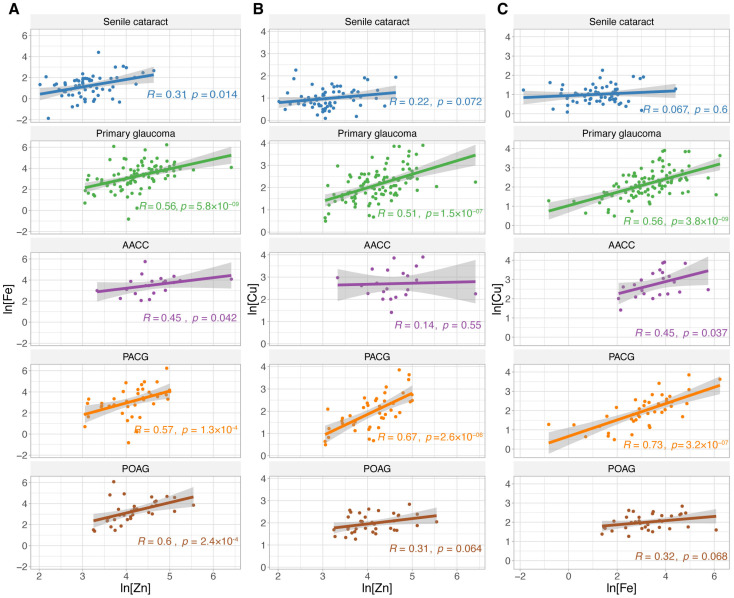
Correlation between trace metal concentrations in the aqueous humor. The natural logarithmic conversion of the trace metal concentrations was performed. The Spearman correlations between (**A**) ln[Zn] and ln[Fe]; (**B**) ln[Zn] and ln[Cu]; and (**C**) ln[Cu] and ln[Fe]. Correlation coefficient R and *p*-values are shown for each group. Abbr.: AACC = acute angle-closure crisis; PACG = primary angle-closure glaucoma; POAG = primary open-angle glaucoma; ln = natural logarithmic transformation; Zn = zinc; Fe = iron; Cu = copper.

We conducted correlation analyses between Zn, Fe, and Cu levels (Figure 2). Patients with senile cataract showed a positive correlation between Fe and Zn levels. In contrast, in glaucoma patients, all three trace metals were positively associated with each other (R ranged from 0.51 to 0.56, *p* < 0.001). Regarding glaucoma subtypes, patients with AACC exhibited a moderate positive correlation between Zn and Fe and between Fe and Cu (R = 0.45, *p* < 0.05 for both). For patients with PACG, all three trace metals were strongly positively related to each other (R ranged from 0.57 to 0.73, *p* < 0.001). For patients with POAG, a positive association was observed only between Fe and Zn levels (R = 0.6, *p* < 0.001).

Furthermore, we compared the ratios of trace metal concentrations (Table 3). The Zn/Fe ratio of primary glaucoma patients was lower than that of senile cataract patients, with the same trends in AACC, PACG, and POAG subtypes (*p* < 0.001 for primary glaucoma, PACG, and POAG vs. senile cataract; *p* < 0.01 for AACC vs. senile cataract). No difference in the Zn/Cu ratio was observed between groups. Patients with glaucoma (including the PACG and POAG subtypes), except for the AACC subtype, had a higher Fe/Cu ratio than those with senile cataract (*p* < 0.001 for primary glaucoma, PACG, and POAG vs. senile cataract). The ratios of trace metals were not significantly different between subtypes.

### 3.4. Relationship Between Clinical Variables and Trace Metal Concentrations in the AH

**Figure 3:**
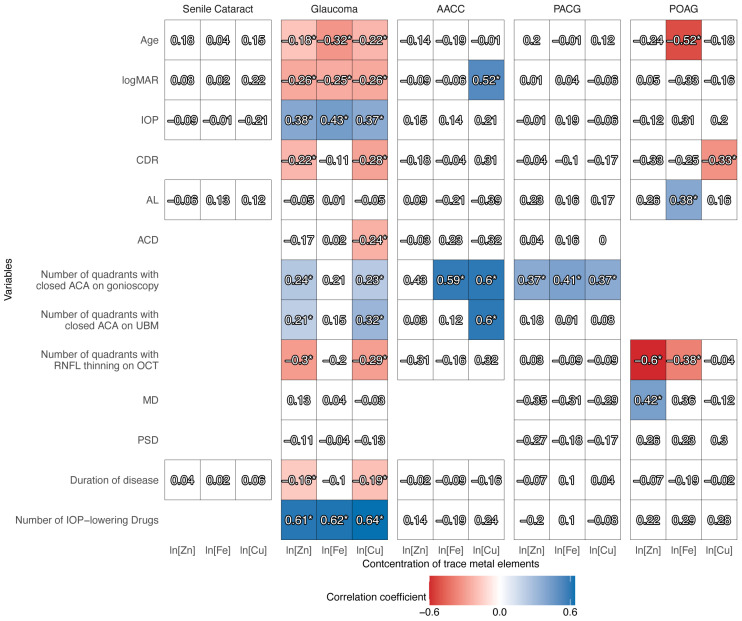
Correlation between trace metal concentrations in the aqueous humor and clinical variables. Spearman correlations. Numbers in cells show the correlation coefficient R. Significant correlations (*p* < 0.05) are marked with “*” and cells are colored. Red and blue indicate negative and positive correlations, respectively. Abbr.: AACC = acute angle-closure crisis; PACG = primary angle-closure glaucoma; POAG = primary open-angle glaucoma; logMAR = logarithm of minimum angle of resolution; IOP = intraocular pressure; CDR = cup-to-disc ratio; AL = axial length; ACD = anterior chamber depth; ACA = anterior chamber angle; UBM = ultrasound biomicroscopy; RNFL = retinal nerve fiber layer; OCT = optical coherence tomography; MD = mean deviation; PSD = pattern standard deviation.

To investigate the clinical factors that affected trace metal levels, we performed Spearman correlation analysis (Figure 3 and Tables S5.1–S5.5). For primary glaucoma, the concentrations of Zn, Fe, and Cu were associated with age, logMAR, IOP, and the number of IOP-lowering drugs. Zn and Cu levels were correlated with CDR, the number of quadrants with closed ACA on gonioscopy, the number of quadrants with closed ACA on UBM, the number of quadrants with RNFL thinning on OCT, and the duration of disease. Cu concentration was also correlated with ACD. In the AACC group, the Fe level was associated with the number of quadrants with closed ACA on gonioscopy, whereas the Cu concentration was related to logMAR, the number of quadrants with closed ACA on gonioscopy, and the number of quadrants with closed ACA on UBM. In the PACG group, the number of quadrants with closed ACA on gonioscopy was associated with all three trace metal levels. In the POAG group, we found the following correlations: Zn level with the number of quadrants with RNFL thinning on OCT and MD; Fe level with age, AL, and the number of quadrants with RNFL thinning on OCT; and Cu level with CDR. The correlations between the variables in different groups are shown in Figures S2.1–S2.5.

We performed multivariate linear regression analysis to identify the most related factors (Table 4 and Figure S3). For the primary glaucoma group, the duration of disease was negatively associated with the concentration of Zn (β = −0.056); the CDR and duration of disease were negatively related to the concentration of Cu (β = −1.033 for CDR, β = −0.063 for duration of disease). The number of quadrants with closed ACA on gonioscopy was positively associated with Fe and Cu levels in the AACC group (β = 0.262 for Fe and β = 0.223 for Cu) and with Zn and Cu levels in the PACG group (β = 0.197 for Zn and β = 0.268 for Cu). In the POAG group, Zn level was negatively associated with the number of quadrants with RNFL thinning on OCT (β = −0.244); Fe level was negatively correlated with age (β = −0.035); and Cu level was negatively associated with CDR (β = −1.456).

### 3.5. Analysis of Potential Biomarkers for the Discrimination of Primary Glaucoma

Multivariate logistic regression analysis was used to assess the potential of trace metals as biomarkers. Zn, Fe, and Cu concentrations in the AH were positively associated with glaucoma diagnosis (Table S6). Among the three primary glaucoma subtypes, the highest odds were found in the AACC group when the AH levels of Zn (OR = 1.096), Fe (OR = 1.129), and Cu (OR = 2.503) were elevated.

**Figure 4:**
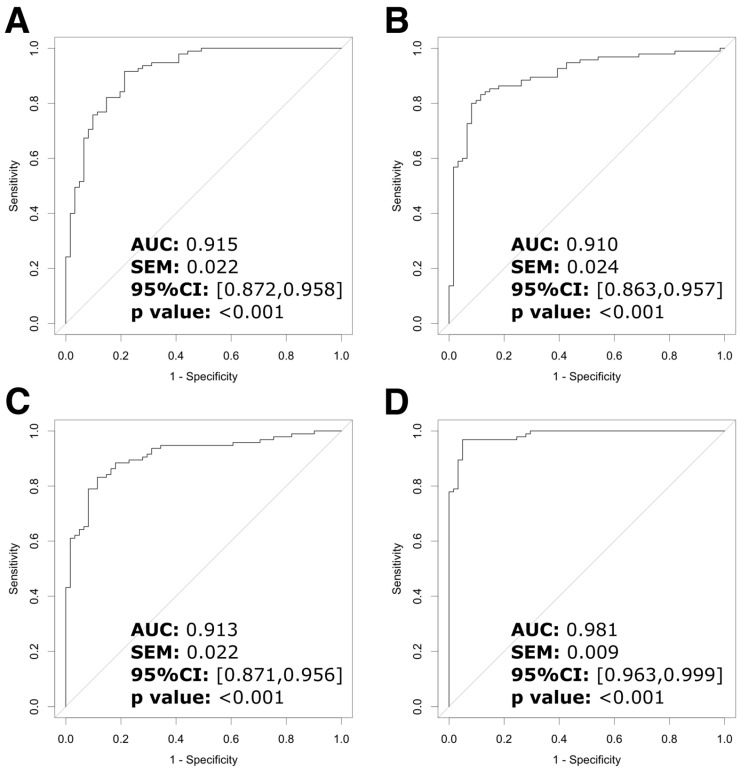
Trace metals as potential biomarkers for discrimination between primary glaucoma and senile cataract. The ROC curves of trace metals in the aqueous humor as biomarkers of primary glaucoma. (**A**) Zn. (**B**) Fe. (**C**) Cu. (**D**) Combination of Zn, Fe, Cu, and confounding variables (age, logMAR, and IOP). Abbr.: ROC = receiver operating characteristic; IOP = intraocular pressure; Zn = zinc; Fe = iron; Cu = copper; AUC = area under the ROC curve; SEM = standard error of the mean; CI = confidence interval.

The predictive values for Zn (AUC = 0.915), Fe (AUC = 0.910), and Cu (AUC = 0.913) were significant (Figure 4A–C). Combining the three trace metals and confounding variables resulted in a higher AUC (0.981, Youden index = 0.921, sensitivity = 0.921, and specificity = 0.951) than using any trace metal alone (Figure 4D). Table 5 displays the thresholds of the trace metals, indicating the highest efficiency in discriminating glaucoma. When the Zn, Fe, and Cu levels in the AH were higher than 30.31, 11.84, and 3.93 μg/L, respectively, patients presented a higher probability of developing primary glaucoma.

## 4. Discussion

This study demonstrates higher AH levels of Zn, Fe, and Cu across all primary glaucoma subtypes, with the highest Zn and Cu concentrations observed in AACC patients (Figure 1).

Akyol et al. [30] revealed higher Cu but stable Zn levels in the AH of patients with glaucoma via atomic absorption spectrometry (AAS). In contrast, Hohberger et al. [20] observed higher Zn and unchanged Cu levels in the AH of patients with POAG using the FI-ICP-MS detection method. Aranaz et al. [21] did not find significant differences in Zn, Fe, and Cu between POAG and controls, as measured by ICP-MS. In line with our results, Bocca et al. [22] reported elevated Fe and Zn levels in the AH of patients with POAG compared with controls, and NikhalaShree et al. [23] revealed elevated Cu concentration in the AH of patients with POAG and PACG.

The pronounced elevation in Zn, Fe, and Cu concentrations in the AACC may be due to the acute interruption of AH drainage. Patients with high IOP showed higher Zn, Fe, and Cu levels than those with low IOP (Figure S1), which was eliminated after adjusting for glaucoma subtype (Table S4). AH retention and increased IOP disrupt the balance of trace metals and temporarily interrupt blood perfusion, leading to cellular dysfunction. Zn accumulation has been observed in experimental pressure-induced retinal ischemia, accompanied by acidosis, oxidative stress, and mitochondrial dysfunction [31,32]. Furthermore, disturbances in Fe homeostasis and ferroptosis caused by pressure-induced glaucoma have recently attracted increasing attention [14,33].

The imbalance of Zn, Fe, and Cu plays critical roles in oxidative stress, structural remodification, and direct neurotoxicity. Dammak et al. [34] observed a significant increase in the levels of oxidative and inflammatory biomarkers in the AH of patients with POAG.

Zn is essential in several molecules and enzymes, such as MMPs, and participates in many pathways. Dysregulation of Zn potentially influences the intraocular homeostasis. AACC patients have higher MMP2 levels in AH [35]. MMPs can decrease the deposition of ECM proteins and facilitate the AH outflow [36]. However, in dexamethasone-induced glaucoma, the dysfunction of TM cells exhibits impaired extracellular Zn^2+^ uptake and decreased intracellular Zn^2+^ [3]. Moreover, Zn^2+^ increases rapidly after optic nerve injury and inhibits the RGC survival and axon regeneration, indicating the critical role of Zn^2+^ in glaucoma retinopathy [37]. The overload of Zn increases lipid peroxidation of retinal pigment epithelial (RPE) cells and induces apoptosis of RPE cells and photoreceptors [38].

Disturbance of Fe homeostasis is related to various ocular diseases. Patients with POAG are associated with high serum iron status indicators (ferritin) and low total iron binding capacity, indicating the possible association between Fe and glaucoma [39]. In addition, the plasma lactoferrin levels are significantly associated with glaucoma severity [40]. A regulatory loop involving Fe homeostasis, transforming growth factor-β2 (TGF-β2), reactive oxygen species (ROS), and ECM may explain the prominent elevation of Fe in the AH and its effects on IOP increase, inflammation, and dysfunction of TM cells [14,41]. Dysregulation of Fe metabolism potentially induces oxidative damage to DNA, proteins, and lipids, and strengthens the oxidative stress in retinal neurodegenerative diseases [5]. Increased IOP can induce accumulation of Fe^2+^ in the retina, especially in the RGC layer, and disruption of redox balance, resulting in ferroptosis in RGCs [33].

Copper plays a critical role in inflammation. Free copper ions are highly redox-active and can contribute to tissue damage by catalyzing the generation of ROS [42]. A high dose of intraocular Cu can induce oxidative stress, hydroxyl radicals generation, DNA strand breaks, lipid peroxidation, and a strong inflammatory reaction [43]. Pretreatment copper chelation can minimize retinal inflammation secondary to laser photocoagulation [44]. The ocular inflammation in return reduces Cu elimination and facilitates Cu redistribution to AH [45]. Topical steroid treatments can reduce the concentration of Cu in the AH [46]. Lysyl oxidase (LOX), a Cu-dependent amine oxidase, can initiate the covalent crosslinking of collagen and elastin and is associated with ECM remodeling [47]. Lysyl oxidase like-2 (LOXL2) is a candidate susceptibility gene for population-specific genetic risk of POAG [48]. The increase of LOXL2 and the activity of LOX in AH has been reported in POAG and PACG patients [23,49].

Regarding the association between Zn, Fe, and Cu, we found a positive correlation between any pair of them, a decreased Zn/Fe ratio, an increased Fe/Cu ratio, and an unchanged Zn/Cu ratio in primary glaucoma and its subtypes (Figure 2 and Table 3), indicating a distinct elevation of Fe. In agreement with us, Bocca et al. [22] found a positive correlation between Cu and Fe levels in patients with POAG. However, Hohberger et al. [20] reported insignificant differences in the ratios of Cu/Zn, Cu/Fe, and Fe/Zn between POAG and controls.

In the pathogenesis of glaucomatous neuropathy, Fe metabolism plays a critical role in regulating inflammation, oxidative stress, mitochondrial dysfunction, cell death, and gene mutations [50]. Characterized by excessive iron-mediated lipid peroxidation and oxidative stress, ferroptosis has been widely reported in neurodegenerative diseases [51] and ocular diseases [52]. In glaucoma, increased IOP can disrupt iron homeostasis, resulting in an excessive accumulation of iron ions (Fe^2+^) in the retina, retinal redox imbalance, and ferroptosis in RGCs [33]. In addition to the metabolism disturbance of Zn and Cu [53], a prominent increase of Fe underscores a strong association between Fe and glaucoma.

Detecting the concentrations of trace metals in microsamples is challenging. Different detection methods could contribute to the inconsistent results between studies. Most researchers have used anodic stripping voltammetry [54] and AAS [23,30] in previous studies. ICP-MS is one of the most powerful techniques for ultra-trace elemental analysis. In our study, by introducing a metal-free microflow sample injection system into the ICP-MS system, FI-ICP-MS can achieve high accuracy and stable analysis of trace metals in micro-samples [55].

In AACC and PACG, the only influencing factor was the number of quadrants with closed ACA on gonioscopy (Table 4). Closed anterior chamber angle displays multiple structural and functional changes. Constant contact of the TM with other structures can injure and obstruct the Schlemm’s canal. The mitochondrial dysfunction in TM cells and the fusion and enlargement of trabecular lamellae sustain the IOP elevation even after relieving the blockage [56]. The progressive angle closure observed with the increasing PACG severity can potentially promote the accumulation of trace metals in AH.

In POAG, the situation can be more complex. Negative associations have been observed between Zn and the number of quadrants with RNFL thinning on OCT, Fe and age, and Cu and the CDR (Table 4). As most patients with POAG remained asymptomatic for a long time, they were diagnosed with moderate or advanced glaucoma owing to late hospitalization.

The anterior structural changes in POAG patients were highly related to the stages. In the early stage, the structural changes are limited to the uveal meshwork [57]. In advanced-stage, structure changes include prominent atrophy of uveal meshwork, adjacent ciliary muscle and iris root, and obliteration of the Schlemm’s canal [57]. Moreover, the increasing amount of sheath-derived plaques in the inner and outer wall of the Schlemm’s canal is negatively correlated with optic nerve axon accounts [58] and optic nerve damage [59]. Advanced pathological changes also involve loss of TM cells together with trabecular lamellae fusion and thickening, cytoskeletal changes in connective tissue cells and ECM remodeling, TGF-β-related matrix component accumulation, and TM cell dysfunction caused by oxidative stress [60].

Moreover, in chronic glaucoma, posterior structural changes include deprivation of neurons and glial cells, thinning of the retina, and loss of optic nerve axons. During glaucoma, neuronal injury can be reflected by a decrease in essential components, including Zn, Fe, and Cu ions, and metal-containing proteins, which are necessary for the physiological function of retinal neurons [16].

In addition, IOP, aqueous dynamics, and cellular metabolism tended to be stable. Thus, a compensatory mechanism may exist; however, this requires further investigation.

Meanwhile, potential confounding factors warrant careful consideration. Although we excluded patients with ocular/systemic diseases, previous trauma/surgeries, medications, or nutritional conditions potentially influencing the concentration of trace metals (please find details in Section 2.2), unanticipated confounding factors may exist.

Regarding demographics, significant intergroup differences in age and sex were observed in the overall population. However, partial correlation analyses revealed no significant association between either age or sex and trace metal concentrations (Tables S1 and S2). To further validate this, we conducted identical statistical analyses on an age- and sex- matched cohort (Tables S7–S11, Figures S4–S7). These analyses yielded results and trends consistent with the overall population, although statistical significance weakened or disappeared for some associations.

The glaucoma severity may influence the trace metal levels in AH. To assess this, we staged glaucoma patients according to the ICD-10 glaucoma stage definition. Due to incomplete visual field (VF) data, particularly among AACC patients, only 52 PACG or POAG patients (10 mild stage, 42 moderate to severe stage) were included in this severity analysis. The number of cases and the baseline characteristics were detailed in Tables S12 and S13. However, no significant difference in Zn, Fe, or Cu levels was observed between the mild and the moderate/severe stage (Figure S8), indicating that the glaucoma severity is not a significant confounder of trace metal levels.

Medication represents another significant confounder. We documented both the number and types of IOP-lowering medications (Table S3). Given the limited sample size for individual drug categories, only the number of IOL-lowering drugs was included in the analyses. We found that the AACC group used less travoprost and more mannitol than the POAG group; the PACG group used less pilocarpine, bimatoprost, and travoprost, and more mannitol than the POAG group. These patterns are clinically plausible: AACC/PACG patients typically require fewer prostaglandin analogs but more osmotic agents like mannitol than POAG patients. Regarding pilocarpine utilization, its higher frequency in POAG versus PACG likely reflects the difference in surgical choices. As PACG patients frequently undergo lens-related surgeries (phacoemulsification with IOL implantation or cataract-glaucoma surgery), pilocarpine is rarely needed preoperatively. Conversely, POAG patients do not require lens-related surgeries and commonly use pilocarpine preoperatively.

While carbonic anhydrase is a zinc-dependent metalloenzyme, the potential impact of carbonic anhydrase inhibitors on Zn concentration in AH remains unclear. According to the describing information of the drugs, Brinzolamide is described chemically as C_12_H_21_N_3_O_5_S_3_ [61], and the chemical formula of methazolamide is C_5_H_8_N_4_O_3_S_2_ [62]. Neither brinzolamide nor methazolamide contains intrinsic trace metal atoms. The sulfonamide group of brinzolamide/methazolamide fits deeply into the active site of carbonic anhydrase, where the negatively charged nitrogen coordinates the zinc ion and sulfonamide group interacts with various key residues, inhibiting the catalytic action [63]. We found no published evidence suggesting direct links between brinzolamide/methazolamide with free zinc ions. Similarly, none of the other medications (timolol, carteolol, brimonidine, pilocarpine, bimatoprost, travoprost, latanoprost, tafluprost, mannitol) contain trace metals. Future studies with larger cohorts are needed to rigorously investigate potential associations between specific IOP-lowering drugs and trace metal concentrations in AH.

Considering the vital role of trace metals in the nervous system [64], elevated Zn, Fe, and Cu levels in the early stages may be accompanied by aggravated glaucomatous damage. In contrast, due to neural signaling deficiencies and retinal tissue deprivation, trace metals may decrease in advanced stages. Young pre-glaucomatous mice show higher concentrations of Zn, Fe, and Cu than aged glaucomatous mice, whereas the latter exhibit lower levels of Zn, Fe, and Cu than age-matched normal mice [65].

We found that Zn, Fe, and Cu can be strong biomarkers to discriminate between primary glaucoma and senile cataract. The combination of the three trace metals performed better than either element alone (Table S5 and Table 5, and Figure 4). It reflected the disturbance of trace metal element homeostasis and can be used in experimental models. Although AH sampling is invasive and may not be directly applied in clinical practice, it indicates the potential of trace metals in AH for discrimination. More convenient and precise sampling and detection methods could achieve this goal in the future.

Moreover, our results revealed the crucial roles of Zn, Fe, and Cu in glaucoma pathology. Trace metal chelation treatment has been proven to be a promising therapeutic strategy for glaucoma. Zn^2+^ chelators reduce RGC death and promote optic nerve regeneration in mouse glaucomatous neuropathy models [37,66]. Fe chelation is effective for retinal degeneration in rat and rabbit models [67]. Oral administration of Fe chelators reduces RGC loss in a mouse glaucoma model [68]. With the development of drug delivery systems, a long-acting injectable thermogel with strong antioxidant activity and drug encapsulation/release efficiency has been reported in glaucoma treatment [69]. Trace metal chelator-loaded systems can be a promising research direction in the future [70].

This study has a large sample size of primary glaucoma patients and controls, including detailed diagnostic categories of AACC, PACG, and POAG. In addition, we collected various clinical variables and investigated the relationships between Zn, Fe, Cu, and clinical factors. However, this study still has some limitations. First, a selection bias existed in the glaucoma patients. As enrollment was restricted to patients undergoing surgery, patients with medically controlled glaucoma and not requiring surgical intervention were excluded. Second, we detected metal concentrations only in the AH, but not in other biosamples such as serum and tears, which should be included in future studies. Third, we only included the numbers of IOP-lowering drugs in our analyses. The types of IOP-lowering drugs are summarized in Table S3, but larger cohorts are necessary to investigate the effects of specific IOP-lowering drugs. Therefore, subsequent research should focus on elucidating the effects of specific medication types, evaluating trace metal concentrations across different ocular tissues, and assessing the therapeutic efficacy of chelation strategies for glaucoma.

## 5. Conclusions

This study investigated the Zn, Fe, and Cu concentrations in AH of patients with glaucoma. Zn, Fe, and Cu concentrations were higher in patients with primary glaucoma than those in patients with senile cataract. Zn and Fe levels in the AACC group were significantly increased compared with those in the PACG and POAG groups. In patients with primary glaucoma, trace metal levels positively correlated mutually, with decreased Zn/Fe and increased Fe/Cu ratios. The number of quadrants with closed anterior chamber angle on gonioscopy was positively associated with Fe and Cu levels in the AACC group and with Zn and Cu levels in the PACG group. In the POAG group, we found negative associations between Zn and the number of quadrants with retinal nerve fiber layer thinning on OCT, Fe and age, and Cu and the cup-to-disc ratio. Trace metals showed high efficiency in discriminating between primary glaucoma and senile cataract.

## 6. Patents

A Chinese patent (#ZL 2020 1 0060253.3) is related to this study.

## Figures and Tables

**Table 1:** Participant demographics and clinical characteristics.

Characteristics	Senile Cataract	Primary Glaucoma				*p* Value *
		Total	AACC	PACG	POAG	
Number of cases, n	67	100	22	42	36	
Sex, male/female	32/35 ^†^	58/42	7/15 ^‡^	18/24 ^§^	33/3 ^†,‡,§^	<0.001
Age, median (IQR), y	67.00 (63.00, 72.00) ^†^	61.00 (50.00, 67.00)	60.50 (53.75, 68.75)	64.00 (60.25, 68.75) ^¶^	52.00 (40.75, 61.25) ^†,¶^	<0.001
logMAR, median (IQR)	1.00 (0.52, 1.30) ^†,¶,^**	0.40 (0.22, 0.82)	0.40 (0.30, 0.67) **	0.40 (0.20, 0.82) ^†^	0.46 (0.22, 1.00) ^¶^	<0.001
IOP, median (IQR), mmHg	12.70 (11.00, 15.00) ^†,‡,§^	20.30 (15.00, 27.25)	18.05 (12.25, 34.67) ^†^	21.85 (17.08, 28.37) ^‡^	19.45 (14.38, 24.18) ^§^	<0.001
CDR, median (IQR)	-	0.80 (0.70, 0.90)	0.50 (0.30, 0.70) ^†,¶^	0.80 (0.70, 0.90) ^¶,^**	0.90 (0.80, 0.91) ^†,^**	<0.001
AL, median (IQR), mm	23.14 (22.76, 23.90) ^†,¶^	23.07 (22.46, 23.88)	22.65 (22.24, 23.18) ^‡^	22.58 (22.05, 23.07) ^†,§^	23.98 (23.66, 25.58) ^‡,§,¶^	<0.001
ACD, median (IQR), mm	-	2.13 (1.90, 2.76)	1.74 (1.56, 1.91) ^†^	2.02 (1.90, 2.34) ^†^	-	<0.001
Number of quadrants with closed ACA on gonioscopy, median (IQR)	-	0.50 (0.00, 3.00)	3.00 (0.50, 4.00)	2.00 (2.00, 3.00)	-	0.564
Number of quadrants with closed ACA on UBM, median (IQR)	-	2.00 (0.00, 3.00)	4.00 (2.25, 4.00) ^†^	3.00 (2.00, 3.00) ^†^	-	0.023
Number of quadrants with RNFL thinning on OCT, median (IQR)	-	2.00 (0.38, 3.50)	0.00 (0.00, 0.00) ^†,‡^	2.00 (1.00, 3.50) ^†^	3.00 (2.00, 4.00) ^‡^	<0.001
MD, median (IQR), dB	-	−21.37 (−28.41, −7.02)	-	−21.37 (−28.16, −16.91)	−26.44 (−30.27, −16.24)	0.305
PSD, median (IQR), dB	-	6.20 (2.26, 9.70)	-	8.09 (2.89, 10.76)	7.30 (2.44, 9.91)	0.628
Duration of disease, median (IQR), y	1.00 (1.00, 2.00) ^†^	1.00 (0.40, 3.00)	0.08 (0.00, 0.44) ^†,‡,§^	1.00 (0.50, 5.00) ^‡^	2.00 (0.88, 3.00) ^§^	<0.001
Number of IOP-lowering drugs, median (IQR)	-	4.00 (2.00, 4.00)	3.50 (2.00, 4.75)	3.00 (1.25, 4.00)	4.00 (2.00, 4.25)	0.227

AACC, PACG, and POAG are subtypes of primary glaucoma. Median (IQR). * Chi-square and Bonferroni adjustment comparisons among groups for variable sex, and Kruskal–Wallis and pairwise comparisons among groups for other variables. For pairwise comparisons between the glaucoma subtypes and the senile cataract group, values in rows sharing the same superscript ^†^: *p* < 0.001, ^‡^: *p* < 0.001, ^§^: *p* < 0.001, ^¶^: *p <* 0.01, **: *p* < 0.05. Abbr.: AACC = acute angle-closure crisis; PACG = primary angle-closure glaucoma; POAG = primary open-angle glaucoma; logMAR = logarithm of minimum angle of resolution; IOP = intraocular pressure; CDR = cup-to-disc ratio; AL = axial length; ACD = anterior chamber depth; ACA = anterior chamber angle; UBM = ultrasound biomicroscopy; RNFL = retinal nerve fiber layer; OCT = optical coherence tomography; MD = mean deviation; PSD = pattern standard deviation.

**Table 2:** Distribution of trace metal concentrations and their natural logarithmic transformation.

Trace Metal	Percentile	Senile Cataract	Primary Glaucoma			
			Total	AACC	PACG	POAG
Zn (μg/L)	0%	7.54	21.14	27.99	21.14	25.76
	25%	16.31	43.99	76.92	41.33	40.36
	50%	21.23	66.80	93.17	66.14	58.28
	75%	28.78	100.89	133.98	90.03	98.14
	100%	102.78	610.35	610.35	148.30	255.70
ln[Zn]	0%	2.02	3.05	3.33	3.05	3.25
	25%	2.79	3.78	4.34	3.72	3.70
	50%	3.06	4.20	4.53	4.19	4.07
	75%	3.36	4.61	4.90	4.50	4.59
	100%	4.63	6.41	6.41	5.00	5.54
Cu (μg/L)	0%	1.09	1.64	4.10	1.64	3.54
	25%	1.98	5.38	9.50	4.07	5.39
	50%	2.51	8.09	14.45	6.85	7.15
	75%	3.54	12.19	26.52	11.86	10.26
	100%	9.58	49.39	49.39	46.95	17.07
ln[Cu]	0%	0.09	0.49	1.41	0.49	1.27
	25%	0.69	1.68	2.25	1.40	1.69
	50%	0.92	2.09	2.67	1.92	1.97
	75%	1.26	2.50	3.28	2.47	2.33
	100%	2.26	3.90	3.90	3.85	2.84
Fe (μg/L)	0%	0.155	0.44	7.82	0.44	3.96
	25%	1.77	14.24	20.07	13.56	12.23
	50%	3.74	27.44	42.78	26.16	24.81
	75%	6.63	53.20	57.55	53.20	46.77
	100%	81.78	509.75	314.11	509.75	436.35
ln[Fe]	0%	−1.87	−0.82	2.06	−0.82	1.38
	25%	0.57	2.66	3.00	2.61	2.50
	50%	1.32	3.31	3.76	3.26	3.21
	75%	1.89	3.97	4.05	3.97	3.85
	100%	4.40	6.23	5.75	6.23	6.08

AACC, PACG, and POAG are subtypes of primary glaucoma. Abbr.: AACC = acute angle-closure crisis; PACG = primary angle-closure glaucoma; POAG = primary open-angle glaucoma; ln = natural logarithmic transformation; Zn = zinc; Fe = iron; Cu = copper.

**Table 3:** Ratios of trace metal concentrations in AH and the difference between patients.

Ratios	Senile Cataract	Primary Glaucoma				*p* Value *
		Total	AACC	PACG	POAG	
Zn/Fe	6.07 (9.77) ^†,‡,§^	2.61 (3.00)	2.86 (3.00) ^§^	2.57 (2.80) ^†^	2.72 (3.03) ^‡^	<0.001
Zn/Cu	8.38 (6.49)	8.19 (7.24)	6.33 (8.02)	9.03 (7.32)	8.27 (6.09)	0.456
Fe/Cu	1.15 (1.84) ^†,‡^	2.78 (4.48)	1.95 (3.26)	3.40 (3.82) ^†^	3.59 (4.84) ^‡^	<0.001

AACC, PACG, and POAG are subtypes of primary glaucoma cases. Median (IQR). * Kruskal–Wallis and Dunnett T3 post hoc comparisons among groups. For pairwise comparisons between the glaucoma subtypes and the senile cataract group, values in rows sharing the same superscript ^†^: *p* < 0.001, ^‡^: *p* < 0.001, ^§^: *p* < 0.01. Abbr.: AH = aqueous humor; AACC = acute angle-closure crisis; PACG = primary angle-closure glaucoma; POAG = primary open-angle glaucoma; Zn = zinc; Fe = iron; Cu = copper.

**Table 4:** Linear regression analysis of trace metal concentrations in the aqueous humor and clinical variables.

Concentration of Metals	Regression Coefficient				Model Summary		
	Variable	Coefficient		*p* Value	Adjusted R^2^	*p* Value	N
		B	SEM				
Primary Glaucoma *							
ln[Zn]	Constant	4.225	0.080	<0.001	0.070	0.017	67
	Duration of disease	−0.056	0.023	0.017			
ln[Cu]	Constant	2.956	0.309	<0.001	0.163	0.001	68
	CDR	−1.033	0.386	0.009			
	Duration of disease	−0.063	0.027	0.024			
AACC *							
ln[Fe]	Constant	2.884	0.288	<0.001	0.291	0.022	15
	Number of quadrants with closed ACA on gonioscopy	0.262	0.101	0.022			
ln[Cu]	Constant	2.467	0.240	<0.001	0.303	0.020	15
	Number of quadrants with closed ACA on gonioscopy	0.223	0.084	0.020			
PACG *							
ln[Zn]	Constant	3.645	0.226	<0.001	0.105	0.037	33
	Number of quadrants with closed ACA on gonioscopy	0.197	0.091	0.037			
ln[Cu]	Constant	1.256	0.293	<0.001	0.116	0.030	33
	Number of quadrants with closed ACA on gonioscopy	0.268	0.117	0.030			
POAG *							
ln[Zn]	Constant	4.812	0.265	<0.001	0.215	0.011	25
	Number of quadrants with RNFL thinning on OCT	−0.244	0.089	0.011			
ln[Fe]	Constant	5.036	0.625	<0.001	0.227	0.007	27
	Age	−0.035	0.012	0.007			
ln[Cu]	Constant	3.258	0.540	<0.001	0.118	0.023	36
	CDR	−1.456	0.610	0.023			

Multivariate linear regression analysis shows the variables included in the best-fitting model, which was selected stepwise in the backward direction according to the Bayesian information criterion. * The best-fitting models are shown in the primary glaucoma and glaucoma subtypes. Abbr.: SEM = standard error of the mean; AACC = acute angle-closure crisis; PACG = primary angle-closure glaucoma; POAG = primary open-angle glaucoma; CDR = cup-to-disc ratio; ACA = anterior chamber angle; RNFL = retinal nerve fiber layer; OCT = optical coherence tomography; Zn = zinc; Fe = iron; Cu = copper.

**Table 5:** Trace metal thresholds for the discrimination of primary glaucoma in aqueous humor.

Trace Metal	Threshold [X] (ln[X]) (μg/L)	Youden Index	Sensitivity (%)	Specificity (%)	PPV (%)	NPV (%)
Zn	30.31 (3.412)	0.691	0.916	0.776	0.867	0.852
Fe	11.84 (2.471)	0.735	0.810	0.919	0.944	0.740
Cu	3.93 (1.370)	0.715	0.879	0.836	0.895	0.812

X: Trace Element. Abbr.: PPV = positive predictive value; NPV = negative predictive value; Zn = zinc; Fe = iron; Cu = copper.

## Data Availability

The data presented in this study are available on request from the corresponding author due to privacy concerns.

## Supplementary Materials

The following supporting information can be downloaded at: https://www.mdpi.com/article/10.3390/biom15070962/s1, Figure S1: Association between trace metal concentrations in the aqueous humor and IOP levels; Figure S2.1: Spearman correlation between clinical variables of patients with senile cataract (control); Figure S2.2: Spearman correlation between clinical variables of patients with primary glaucoma; Figure S2.3: Spearman correlation between clinical variables of patients with AACC; Figure S2.4: Spearman correlation between clinical variables of patients with PACG; Figure S2.5: Spearman correlation between clinical variables of patients with POAG; Figure S3: Relationship between trace metals in the aqueous humor and clinical variables in the best regression model; Figure S4: The trace metal concentrations in the aqueous humor of primary glaucoma patients and controls for age- and sex-matched participants; Figure S5: Correlation between trace metal concentrations in the aqueous humor for age- and sex-match participants; Figure S6: Correlation between trace metal concentrations in the aqueous humor and clinical variables for age- and sex-match participants; Figure S7: Trace metals as potential biomarkers for discrimination between primary glaucoma and senile cataract for age- and sex-matched participants; Figure S8: The trace metal concentrations in the aqueous humor by glaucoma stage; Table S1: Relationship between age and trace metal concentrations; Table S2: Relationship between sex and trace metal concentrations; Table S3: The types of IOP-lowering drugs; Table S4: Partial correlation analysis for IOP and trace metal concentrations adjusted by diagnostic group; Table S5.1: Univariate correlations between clinical variables and trace metal concentrations in aqueous humor of patients with senile cataract; Table S5.2: Univariate correlations between clinical variables and trace metal concentrations in aqueous humor of patients with primary glaucoma; Table S5.3: Univariate correlations between clinical variables and trace metal concentrations in aqueous humor of patients with AACC; Table S5.4: Univariate correlations between clinical variables and trace metal concentrations in aqueous humor of patients with PACG; Table S5.5: Univariate correlations between clinical variables and trace metal concentrations in aqueous humor of patients with POAG; Table S6: Association between glaucoma subtypes and trace metal concentrations in aqueous humor; Table S7: Age- and sex-matched participant demographics and clinical characteristics; Table S8: Distribution of trace metal concentrations and their natural logarithmic transformation for age- and sex-matched participants; Table S9: Ratios of trace metal concentrations in AH and the difference between patients for age- and sex-matched participants; Table S10: Linear regression analysis of trace metal concentrations in the aqueous humor and clinical variables for age- and sex-matched participants; Table S11: Trace metal thresholds for the discrimination of primary glaucoma in aqueous humor for age- and sex-matched participants; Table S12: Number of cases stratified by glaucoma stage; Table S13: Participant demographics and clinical characteristics by glaucoma stage.

## Abbreviations

The following abbreviations are used in this manuscript:

RGCs	Retinal ganglion cells
IOP	Intraocular pressure
TM	Trabecular meshwork
PACG	Primary angle-closure glaucoma
POAG	Primary open-angle glaucoma
AACC	Acute angle-closure crisis
Zn	Zinc
Fe	Iron
Cu	Copper
MMPs	Matrix metalloproteinases
ECM	Extracellular matrix
NMDA	N-methyl-D-aspartic acid
AMPA	α-amino-3-hydroxy-5-methyl-4-isoxazolepropionic acid
AH	Aqueous humor
FI-ICP-MS	Flow injection inductively coupled plasma mass spectrometry
VA	Visual acuity
CDR	Cup-to-disc ratio
logMAR	Logarithm of minimum angle of resolution
AL	Axial length
RNFL	Retinal nerve fiber layer
OCT	Optical coherence tomography
ACA	Anterior chamber angle
UBM	Ultrasound biomicroscopy
ACD	Anterior chamber depth
MD	Mean deviation
PSD	Pattern standard deviation
VF	Visual field
HNO_3_	Nitric acid
Ge	Germanium
Sc	Scandium
In	Indium
ICP-MS	Inductively coupled plasma mass spectrometer
ln	Natural logarithmic transformation
ROC	Receiver operating characteristic
AUC	Area under the ROC curve
ORs	Odds ratios
AAS	Atomic absorption spectrometry
RPE	Retinal pigment epithelial
TGF-β2	Transforming growth factor-β2
ROS	Reactive oxygen species
LOX	Lysyl oxidase
LOXL2	Lysyl oxidase like-2
