# Supplementary material for: Levels of Zinc, Iron, and Copper in the Aqueous Humor of Patients with Primary Glaucoma
Source: Biomolecules. 2025 Jul 4;15(7):962. doi: 10.3390/biom15070962 (PMC12292386; doi:10.3390/biom15070962)
Supplement: Supplementary file 1 [file biomolecules-15-00962-s001.zip › biomolecules-3671387-supplementary.pdf]

Table S1. Relationship between age and trace metal concentrations.

		Zn	Fe	Cu
**Age**	**r**	0.138	-0.088	0.061
	***p* value**	0.129	0.338	0.506

Partial correlation analysis. Controlled variables: IOP. Abbr.: Zn = zinc; Fe = iron; Cu = copper.

Table S2. Relationship between sex and trace metal concentrations.

		Zn	Fe	Cu
**Sex**	**r**	-0.113	-0.016	-0.052
	***p* value**	0.151	0.843	0.504

Partial correlation analysis. Controlled variables: IOP. Abbr.: Zn = zinc; Fe = iron; Cu = copper.

Table S3. The types of IOP-lowering drugs.

Drug	Primary glaucoma				*p* value*
	Total	AACC	PACG	POAG	
Number of cases, n	100	22	42	36	
Brinzolamide, n (%)	62 (62.0%)	14 (63.6%)	24 (57.1%)	24 (66.7%)	0.697
Timolol, n (%)	54 (54.0%)	11 (50.0%)	21 (50.0%)	22 (61.1%)	0.579
Carteolol, n (%)	8 (8.0%)	2 (9.1%)	3 (7.1%)	3 (8.3%)	1
Brimonidine, n (%)	48 (48.0%)	8 (36.4%)	23 (54.8%)	17 (47.2%)	0.382
Pilocarpine, n (%)	55 (55.0%)	12 (54.5%)	16 (38.1%) §	27 (75.0%) §	0.005
Bimatoprost, n (%)	9 (9.0%)	0 (0.0%)	1 (2.4%) ¶	8 (22.2%) ¶	0.002
Travoprost, n (%)	11 (11.0%)	0 (0.0%) †	1 (2.4%) §	10 (27.8%) †,§	<0.001
Latanoprost, n (%)	5 (5.0%)	0 (0.0%)	3 (7.1%)	2 (5.6%)	0.721
Tafluprost, n (%)	2 (2.0%)	1 (4.5%)	1 (2.4%)	0 (0.0%)	0.695
Methazolamide, n (%)	13 (13.0%)	7 (31.8%)	3 (7.1%)	3 (8.3%)	0.021
Mannitol, n (%)	54 (54.0%)	19 (86.4%) ‡	27 (64.3%) **	8 (22.2%) ‡,**	<0.001
Number of IOP-lowering drugs, median (IQR)	4.00 (2.00, 4.00)	3.50 (2.00, 4.75)	3.00 (1.25, 4.00)	4.00 (2.00, 4.25)	0.227

AACC, PACG, and POAG are subtypes of primary glaucoma. n (%) for each drug. Median (IQR) for number of IOP-lowering drugs. * Chi-square and Bonferroni adjustment comparisons among glaucoma subgroups for each drug, and Kruskal–Wallis and pairwise comparisons among glaucoma subgroups for the number of IOP-lowering drugs. For pairwise comparisons between the glaucoma subtypes, values in rows sharing the same superscript †: $p < 0.05$, ‡: $p < 0.001$, §: $p < 0.01$, ¶: $p < 0.05$, **: $p < 0.001$. Abbr.: AACC = acute angle-closure crisis; PACG = primary angle-closure glaucoma; POAG = primary open-angle glaucoma.

Table S4. Partial correlation analysis for IOP and trace metal concentrations adjusted by diagnostic group.

		Zn	Fe	Cu
**IOP**	**r**	-0.027	0.095	0.107
	***p* value**	0.731	0.235	0.170

Partial correlation analysis. Controlled variable: diagnostic groups. Abbr.: Zn = zinc; Fe = iron; Cu = copper.

Table S5.1. Univariate correlations between clinical variables and trace metal concentrations in aqueous humor of patients with senile cataract.

**Variables**	**Baseline**	**ln[Zn]**		**ln[Fe]**		**ln[Cu]**	
		**Correlation Coefficient**	***p* value**	**Correlation Coefficient**	***p* value**	**Correlation Coefficient**	***p* value**
Age (year)	67.00 (9.00)	0.18	0.145	0.04	0.752	0.15	0.239
logMAR	1.00 (0.78)	0.08	0.511	0.02	0.892	0.22	0.077
IOP (mmHg)	12.70 (4.00)	-0.09	0.46	-0.01	0.943	-0.21	0.092
AL (mm)	23.14 (1.21)	-0.06	0.643	0.13	0.333	0.12	0.344
Duration of disease (year)	1.00 (1.00)	0.05	0.719	0.02	0.893	0.07	0.599

Spearman correlation, * *p < 0.05*. Baseline shown as median (IQR). Abbr.: Zn = zinc; Fe = iron; Cu = copper; logMAR = logarithm of minimum angle of resolution; IOP = intraocular pressure; AL = axial length.

Table S5.2. Univariate correlations between clinical variables and trace metal concentrations in aqueous humor of patients with primary glaucoma.

**Variables**	**Baseline**	**ln[Zn]**		**ln[Fe]**		**ln[Cu]**	
		**Correlation Coefficient**	***p* value**	**Correlation Coefficient**	***p* value**	**Correlation Coefficient**	***p* value**
Age (year)	61.11 (17.00)	-0.18	0.024*	-0.32	<0.001*	-0.22	0.004*
logMAR	0.40 (0.60)	-0.26	<0.001*	-0.25	0.001*	-0.26	<0.001*
IOP (mmHg)	20.30 (12.80)	0.38	<0.001*	0.43	<0.001*	0.37	<0.001*
CDR	0.80 (0.20)	-0.22	0.029*	-0.11	0.277	-0.28	0.006*
AL (mm)	23.07 (1.46)	-0.05	0.495	0.01	0.926	-0.05	0.528
ACD (mm)	2.13 (1.00)	-0.17	0.096	0.02	0.881	-0.24	0.02*
Number of quadrants with closed ACA on gonioscopy	0.50 (3.00)	0.24	0.036*	0.21	0.062	0.23	0.04*
Number of quadrants with closed ACA on UBM	2.00 (3.00)	0.21	0.043*	0.15	0.163	0.32	0.001*

Number of quadrants with RNFL thinning on OCT	2.00 (3.00)	-0.3	0.004*	-0.2	0.068	-0.29	0.005*
MD (dB)	-21.37 (22.00)	0.13	0.33	0.04	0.792	-0.03	0.798
PSD (dB)	6.20 (8.00)	-0.11	0.389	-0.04	0.761	-0.13	0.309
Duration of disease (year)	1.00 (2.65)	-0.16	0.043*	-0.1	0.188	-0.19	0.016*
Number of IOP-lowering Drugs	4.00 (2.00)	0.61	<0.001*	0.62	<0.001*	0.64	<0.001*

Spearman correlation, * $p < 0.05$. Baseline shown as median (IQR). Abbr.: Zn = zinc; Fe = iron; Cu = copper; AACC = acute angle-closure crisis; PACG = primary angle-closure glaucoma; POAG = primary open-angle glaucoma; logMAR = logarithm of minimum angle of resolution; IOP = intraocular pressure; CDR = cup-to-disc ratio; AL = axial length; ACD = anterior chamber depth; ACA = anterior chamber angle; UBM = ultrasound biomicroscopy; RNFL = retinal nerve fiber layer; OCT = optical coherence tomography; MD = mean deviation; PSD = pattern standard deviation.

Table S5.3. Univariate correlations between clinical variables and trace metal concentrations in aqueous humor of patients with AACC.

Variables	Baseline	ln[Zn]		ln[Fe]		ln[Cu]	
		Correlation Coefficient	*p* value	Correlation Coefficient	*p* value	Correlation Coefficient	*p* value
Age (year)	60.50 (17.00)	-0.14	0.547	-0.19	0.41	-0.01	0.98
logMAR	0.40 (0.42)	-0.09	0.713	-0.06	0.774	0.52	0.013*
IOP (mmHg)	18.05 (23.30)	0.15	0.503	0.14	0.542	0.21	0.355
CDR	0.50 (0.00)	-0.18	0.456	-0.04	0.855	0.31	0.188
AL (mm)	22.65 (1.02)	0.09	0.703	-0.21	0.348	-0.39	0.069
ACD (mm)	1.74 (0.37)	-0.03	0.915	0.23	0.299	-0.32	0.143
Number of quadrants with closed ACA on gonioscopy	3.00 (4.00)	0.43	0.126	0.59	0.022*	0.6	0.018*
Number of quadrants with closed ACA on UBM	4.00 (2.00)	0.03	0.898	0.12	0.593	0.6	0.003*
Number of quadrants with RNFL thinning on OCT	0.00 (0.00)	-0.31	0.21	-0.16	0.519	0.32	0.187
Duration of disease (year)	0.08 (0.50)	-0.02	0.944	-0.09	0.691	-0.16	0.484

Number of IOP-lowering Drugs	3.50 (3.00)	0.14	0.532	-0.19	0.399	0.24	0.284

Spearman correlation, * $p < 0.05$. Baseline shown as median (IQR). Abbr.: Zn = zinc; Fe = iron; Cu = copper; AACC = acute angle-closure crisis; logMAR = logarithm of minimum angle of resolution; IOP = intraocular pressure; CDR = cup-to-disc ratio; AL = axial length; ACD = anterior chamber depth; ACA = anterior chamber angle; UBM = ultrasound biomicroscopy; RNFL = retinal nerve fiber layer; OCT = optical coherence tomography.

Table S5.4. Univariate correlations between clinical variables and trace metal concentrations in aqueous humor of patients with PACG.

Variables	Baseline	ln[Zn]		ln[Fe]		ln[Cu]	
		Correlation Coefficient	*p* value	Correlation Coefficient	*p* value	Correlation Coefficient	*p* value
Age (year)	64.00 (9.00)	0.2	0.213	-0.01	0.933	0.12	0.468
logMAR	0.40 (0.73)	0.01	0.932	0.04	0.798	-0.06	0.696
IOP (mmHg)	21.85 (11.50)	-0.01	0.961	0.19	0.238	-0.06	0.691
CDR	0.80 (0.20)	-0.04	0.818	-0.1	0.555	-0.17	0.292
AL (mm)	22.58 (1.05)	0.23	0.146	0.16	0.312	0.17	0.294
ACD (mm)	2.02 (0.00)	0.04	0.807	0.16	0.359	0	0.999
Number of quadrants with closed ACA on gonioscopy	2.00 (1.00)	0.37	0.035*	0.41	0.018*	0.37	0.036*
Number of quadrants with closed ACA on UBM	3.00 (2.00)	0.18	0.304	0.01	0.951	0.08	0.657
Number of quadrants with RNFL thinning on OCT	2.00 (3.00)	0.03	0.878	-0.09	0.614	-0.09	0.585
MD (dB)	-21.37 (14.00)	-0.35	0.08	-0.31	0.127	-0.29	0.157
PSD (dB)	8.09 (8.00)	-0.27	0.188	-0.18	0.377	-0.17	0.399
Duration of disease (year)	1.00 (4.50)	-0.07	0.653	0.1	0.538	0.04	0.785
Number of IOP-lowering Drugs	3.00 (3.00)	-0.2	0.21	0.1	0.555	-0.08	0.6

Spearman correlation, * $p < 0.05$. Baseline shown as median (IQR). Abbr.: Zn = zinc; Fe = iron; Cu = copper; PACG = primary angle-closure glaucoma; logMAR = logarithm of minimum angle of resolution; IOP = intraocular pressure; CDR = cup-to-disc ratio; AL = axial length; ACD = anterior chamber depth; ACA = anterior chamber angle; UBM = ultrasound biomicroscopy; RNFL = retinal nerve fiber layer; OCT = optical coherence tomography; MD = mean deviation; PSD = pattern standard deviation.

Table S5.5. Univariate correlations between clinical variables and trace metal concentrations in aqueous humor of patients with POAG.

Variables	Baseline	ln[Zn]		ln[Fe]		ln[Cu]	
		Correlation Coefficient	*p* value	Correlation Coefficient	*p* value	Correlation Coefficient	*p* value
Age (year)	52.00 (24.00)	-0.24	0.164	-0.52	0.002*	-0.18	0.283
logMAR	0.46 (0.79)	0.05	0.775	-0.33	0.057	-0.16	0.346
IOP (mmHg)	19.45 (10.40)	-0.12	0.477	0.31	0.072	0.2	0.234
CDR	0.90 (0.14)	-0.33	0.052	-0.25	0.157	-0.33	0.048*
AL (mm)	23.99 (2.00)	0.26	0.153	0.38	0.038*	0.16	0.385
Number of quadrants with RNFL thinning on OCT	3.00 (2.00)	-0.6	<0.001*	-0.38	0.032*	-0.04	0.815
MD (dB)	-26.44 (15.00)	0.42	0.034*	0.36	0.088	-0.12	0.558
PSD (dB)	7.30 (8.00)	0.26	0.196	0.23	0.285	0.3	0.131
Duration of disease (year)	2.00 (2.38)	-0.07	0.689	-0.19	0.272	-0.02	0.927
Number of IOP-lowering Drugs	4.00 (3.00)	0.22	0.204	0.29	0.1	0.28	0.1

Spearman correlation, * $p < 0.05$. Baseline shown as median (IQR). Abbr.: Zn = zinc; Fe = iron; Cu = copper; POAG = primary open-angle glaucoma; logMAR = logarithm of minimum angle of resolution; IOP = intraocular pressure; CDR = cup-to-disc ratio; AL = axial length; RNFL = retinal nerve fiber layer; OCT = optical coherence tomography; MD = mean deviation; PSD = pattern standard deviation.

Table S6. Association between glaucoma subtypes and trace metal concentrations in aqueous humor.

Reference Category	Outcome Category	No.(%) of Participants	OR (95% CI) of Zn		OR (95% CI) of Fe		OR (95% CI) of Cu	
			Unadjusted	Adjusted†	Unadjusted	Adjusted†	Unadjusted	Adjusted†
Senile Cataract	-	67 (38.5%)‡	*1*	*1*	*1*	*1*	*1*	*1*
	AACC	22 (12.6%)§	1.094 (1.065, 1.124)*	1.096 (1.061, 1.133)*	1.154 (1.094, 1.218)*	1.129 (1.066, 1.195)*	2.454 (1.875, 3.212)*	2.503 (1.775, 3.529)*
	PACG	42 (24.1%)‖	1.078 (1.051, 1.106)*	1.078 (1.045, 1.113)*	1.153 (1.093, 1.217)*	1.128 (1.066, 1.194)*	2.268 (1.738, 2.960)*	2.283 (1.623, 3.211)*
	POAG	36 (20.7%)#	1.079 (1.052, 1.107)*	1.082 (1.048, 1.117)*	1.153 (1.093, 1.217)*	1.127 (1.065, 1.193)*	2.142 (1.643, 2.794)*	2.202 (1.564, 3.100)*
PACG	-	42 (24.1%)‖	*1*	*1*	*1*	*1*	*1*	*1*
	AACC	22 (12.6%)§	1.015 (1.002, 1.027)*	1.016 (1.003, 1.029)*	1.001 (0.994, 1.008)	1.001 (0.994, 1.007)	1.082 (1.023, 1.144)*	1.096 (1.028, 1.168)*
	POAG	36 (20.7%)#	1.001 (0.990, 1.013)	1.003 (0.991, 1.016)	1.000 (0.994, 1.007)	0.999 (0.992, 1.007)	0.945 (0.871, 1.024)	0.965 (0.881, 1.056)
POAG	-	36 (20.7%)#	*1*	*1*	*1*	*1*	*1*	*1*
	AACC	22 (12.6%)§	1.014 (1.001, 1.026)*	1.013 (0.999, 1.027)	1.001 (0.994, 1.008)	1.001 (0.994, 1.009)	1.145 (1.052, 1.247)*	1.136 (1.038, 1.244)*

*: OR with statistical significance. *Italic: reference.* †: adjusted for age, logMAR, and IOP. ‡: Fe missing 5, §: Zn missing 1, ‖: Fe missing 1, #: Fe missing 2. Abbr.: Zn = zinc; Fe = iron; Cu = copper; OR = odds ratio; CI = confidence interval.

Table S7. Age- and sex-matched participant demographics and clinical characteristics.

Characteristics	Senile cataract	Primary Glaucoma				p value[*]
		Total	AACC	PACG	POAG	
Number of cases, n	47	63	17	36	10	
Sex, male/female	20/27	24/39	4/13	13/23	7/3	0.11
Age, median (IQR), y	65.00 (60.00, 68.00)	64.00 (61.00, 69.00)	63.00 (60.00, 70.00)	65.00 (61.00, 69.00)	61.50 (56.00, 66.00)	0.32
logMAR, median (IQR)	1.00 (0.52, 1.22)†,‡	0.40 (0.22, 0.96)	0.40 (0.30, 0.70)†	0.40 (0.22, 0.82)‡	0.35 (0.22, 1.30)	0.003
IOP, median (IQR), mmHg	12.70 (11.00, 14.65)†,§,¶	20.50 (15.20, 28.75)	18.10 (12.00, 36.60)†	21.85 (17.23, 28.42)§	18.75 (13.02, 21.75)¶	<0.001
CDR, median (IQR)	-	0.80 (0.60, 0.90)	0.50 (0.35, 0.70)‡,**	0.80 (0.70, 0.90)‡	0.90 (0.81, 0.90)**	<0.001
AL, median (IQR), mm	23.10 (22.69, 23.85)‡,†	22.79 (22.18, 23.28)	22.59 (22.18, 23.13)§	22.67 (22.01, 23.06)$^{\ddagger,\#}$	23.92 (23.46, 24.13)$^{\dagger,\S,\#}$	<0.001
ACD, median (IQR), mm	-	2.02 (1.81, 2.39)	1.68 (1.50, 1.90)§	2.03 (1.91, 2.35)§	-	<0.001
Number of quadrants with closed ACA on gonioscopy, median (IQR)	-	2.00 (0.00, 3.00)	2.50 (0.75, 4.00)	2.00 (2.00, 3.00)	-	0.729
Number of quadrants with closed ACA on UBM, median (IQR)	-	3.00 (0.00, 3.00)	4.00 (3.00, 4.00)†	3.00 (0.00, 3.00)†	-	0.019
Number of quadrants with RNFL thinning on OCT, median (IQR)	-	2.00 (0.00, 3.00)	0.00 (0.00, 0.25)$^{\S,\#}$	2.00 (1.00, 3.50)§	2.75 (2.38, 3.62)$^{\#}$	<0.001
MD, median (IQR), dB	-	-18.40 (-27.00, -7.13)	-	-20.90 (-27.50, -16.74)	-19.94 (-28.04, -16.83)	0.850
PSD, median (IQR), dB	-	7.93 (3.53, 9.52)	-	8.31 (3.66, 11.03)	7.99 (4.43, 8.65)	0.626
Duration of disease, median (IQR), y	1.00 (1.00, 2.00)§	1.00 (0.50, 4.50)	0.08 (0.00, 0.50)$^{\S,\#,\ddagger}$	1.00 (0.50, 5.25)$^{\#}$	2.50 (2.00, 3.75)‡	<0.001
Number of IOP-lowering drugs, median (IQR)	-	3.00 (1.00, 4.00)	4.00 (2.00, 5.00)	3.00 (1.00, 4.00)	3.50 (1.25, 4.75)	0.435

AACC, PACG, and POAG are subtypes of primary glaucoma. Median (IQR). [*] Chi-square and Bonferroni adjustment comparisons among groups for variable sex, and Kruskal–Wallis and pairwise comparisons among groups for other variables. For pairwise comparisons between the glaucoma subtypes and the senile cataract group, values in rows sharing the same superscript †: $p < 0.05$, ‡: $p < 0.01$, §: $p < 0.001$, ¶: $p < 0.05$, **: $p < 0.01$, $^{\#}$: $p < 0.001$. Abbr.: AACC = acute angle-closure crisis; PACG = primary angle-closure glaucoma; POAG = primary open-angle glaucoma; logMAR = logarithm of minimum angle of resolution; IOP = intraocular pressure; CDR = cup-to-disc ratio; AL = axial length; ACD = anterior chamber depth; ACA = anterior chamber angle; UBM = ultrasound biomicroscopy; RNFL = retinal nerve fiber layer; OCT = optical coherence tomography; MD = mean deviation; PSD = pattern standard deviation.

Table S8. Distribution of trace metal concentrations and their natural logarithmic transformation for age- and sex-matched participants.

Trace metal	Percentile	Senile cataract	Primary Glaucoma			
			Total	AACC	PACG	POAG
Zn (μg/L)	0%	8.66	21.14	27.99	21.14	38.47
	25%	16.31	54.69	67.30	50.73	52.93
	50%	20.16	77.79	85.56	70.74	82.16
	75%	27.05	111.60	133.98	98.20	159.17
	100%	68.38	610.35	610.35	148.30	255.70
ln[Zn]	0%	2.16	3.05	3.33	3.05	3.65
	25%	2.79	4.00	4.21	3.93	3.97
	50%	3.00	4.35	4.45	4.26	4.39
	75%	3.30	4.72	4.90	4.59	5.07
	100%	4.23	6.41	6.41	5.00	5.54
Cu (μg/L)	1.09	1.64	1.09	7.44	1.64	3.54
	1.97	5.71	1.97	9.61	4.50	5.87
	2.48	9.61	2.48	13.64	7.93	7.91
	3.56	14.49	3.56	27.47	12.23	10.55
	9.58	49.39	9.58	49.39	46.95	17.07
ln[Cu]	0%	0.09	0.49	2.01	0.49	1.27
	25%	0.68	1.74	2.26	1.50	1.77
	50%	0.91	2.26	2.61	2.07	2.07
	75%	1.27	2.67	3.31	2.50	2.36
	100%	2.26	3.90	3.90	3.85	2.84
Fe (μg/L)	0%	0.16	0.44	7.82	0.44	5.97
	25%	1.77	15.74	19.93	15.40	13.48
	50%	3.72	28.38	32.06	27.53	34.11
	75%	6.15	50.63	49.26	54.03	45.73
	100%	81.78	509.75	314.11	509.75	107.52
ln[Fe]	0%	-1.87	-0.82	2.06	-0.82	1.79
	25%	0.57	2.76	2.99	2.73	2.58
	50%	1.31	3.35	3.47	3.32	3.50
	75%	1.82	3.92	3.90	3.99	3.82
	100%	4.40	6.23	5.75	6.23	4.68

AACC, PACG, and POAG are subtypes of primary glaucoma. Abbr.: AACC = acute angle-closure crisis; PACG = primary angle-closure glaucoma; POAG = primary open-angle glaucoma; ln = natural logarithmic transformation; Zn = zinc; Fe = iron; Cu = copper.

Table S9. Ratios of trace metal concentrations in AH and the difference between patients for age- and sex-matched participants.

Ratios	Senile cataract	Primary Glaucoma				*p* value*
		Total	AACC	PACG	POAG	
Zn/Fe	5.56(12.38) †, ‡	2.67(3.33)	2.86(3.00) †	2.57(2.80) ‡	3.18(3.41)	**<0.001**
Zn/Cu	7.97(6.31)	8.16(7.63)	5.74(6.83)	9.11(7.34)	11.71(6.31)	0.099
Fe/Cu	1.14(1.86) †, ‡	3.06(4.75)	1.66(1.28)	3.46(4.00) ‡	4.61(3.97) †	**<0.001**

AACC, PACG, and POAG are subtypes of primary glaucoma cases. Median (IQR). * Kruskal–Wallis and Dunnett T3 post hoc comparisons among groups. For pairwise comparisons between the glaucoma subtypes and the senile cataract group, values in rows sharing the same superscript †: *p < 0.01*, ‡: *p < 0.001*. Abbr.: AH = aqueous humor; AACC = acute angle-closure crisis; PACG = primary angle-closure glaucoma; POAG = primary open-angle glaucoma; Zn = zinc; Fe = iron; Cu = copper.

Table S10. Linear regression analysis of trace metal concentrations in the aqueous humor and clinical variables for age- and sex-matched participants.

Concen-tration of metals	Regression Coefficient				Model Summary		
	Variable	Coefficient		*p* value	Adjusted R^2	*p* value	N
		B	SEM				
**Primary Glaucoma** *							
ln[Cu]	Constant	1.885	0.102	<0.001	0.093	0.002	90
	Number of quadrants with closed ACA on UBM	0.137	0043	<0.001			
**AACC** *							
ln[Fe]	Constant	2.884	0.288	<0.001	0.291	0.022	15
	Number of quadrants with closed ACA on gonioscopy	0.262	0.101	0.022			
ln[Cu]	Constant	2.467	0.240	<0.001	0.411	0.003	22
	Number of quadrants with closed ACA on UBM	0.317	0.098	0.004			
	logMAR	0.375	0.165	0.035			
**POAG** *							
ln[Zn]	Constant	4.881	0.226	<0.001	0.281	<0.001	33
	Number of quadrants with RNFL thinning on OCT	-0.276	0.075	<0.001			
ln[Cu]	*Constant*	3.258	0.540	<0.001	0.118	0.023	36
	CDR	-1.456	0.610	0.023			

Multivariate linear regression analysis shows the variables included in the best-fitting model, which was selected stepwise in the backward direction according to the Bayesian information criterion. * The best-fitting models are shown in the primary glaucoma and

glaucoma subtypes. Abbr.: SEM = standard error of the mean; AACC = acute angle-closure crisis; PACG = primary angle-closure glaucoma; POAG = primary open-angle glaucoma; CDR = cup-to-disc ratio; ACA = anterior chamber angle; RNFL = retinal nerve fiber layer; OCT = optical coherence tomography; Zn = zinc; Fe = iron; Cu = copper.

Table S11. Trace metal thresholds for the discrimination of primary glaucoma in aqueous humor for age- and sex-matched participants.

Trace Metal	Threshold [X](ln[X]) (μg/L)	Youden Index	Sensitivity (%)	Specificity (%)	PPV (%)	NPV (%)
Zn	47.28 (3.856)	0.736	0.823	0.913	0.927	0.792
Fe	11.22 (2.418)	0.752	0.839	0.913	0.929	0.808
Cu	5.26 (1.660)	0.741	0.806	0.935	0.943	0.782

X: Trace Element. Abbr.: PPV = positive predictive value; NPV = negative predictive value; Zn = zinc; Fe = iron; Cu = copper.

Table S12. Number of cases stratified by glaucoma stage.

Stage *	Total	PACG	POAG
Mild, n	10	5	5
Moderate, n	1	1	0
Severe, n	41	20	21
Undefined, n	26	16	10

* According to the ICD-10 glaucoma stage definition

Table S13. Participant demographics and clinical characteristics by glaucoma stage.

Characteristics	Total	Mild	Moderate to Severe	*p* value*
Number of cases, n	52	10	42	
Sex, male/female	38/14	5/5	33/9	0.152
Age, median (IQR), y	55.15 (13.60)	51.10 (17.10)	56.12 (12.69)	0.299
logMAR, median (IQR)	0.30 (0.15, 0.70)	0.13 (0.01, 0.26)	0.40 (0.22, 1.00)	0.011
IOP, median (IQR), mmHg	21.35 (15.30, 26.25)	19.65 (17.00, 23.85)	21.85 (15.10, 27.00)	0.693
CDR, median (IQR)	0.80 (0.80,	0.70 (0.62,	0.90 (0.80,	0.002

	0.90)	0.80)	0.90)	
AL, median (IQR), mm	23.59 (22.68, 24.08)	22.79 (22.58, 25.87)	23.59 (22.89, 23.96)	0.757
ACD, mean (SD), mm	2.60 (0.57)	2.57 (0.66)	2.60 (0.55)	0.874
Number of quadrants with closed ACA on gonioscopy, median (IQR)	0.00 (0.00, 2.00)	0.00 (0.00, 1.00)	0.00 (0.00, 2.00)	0.357
Number of quadrants with closed ACA on UBM, median (IQR)	0.00 (0.00, 3.00)	0.00 (0.00, 3.75)	0.00 (0.00, 3.00)	0.654
Number of quadrants with RNFL thinning on OCT, median (IQR)	2.00 (1.25, 4.00)	0.25 (0.00, 1.00)	2.50 (2.00, 4.00)	<0.001
MD, median (IQR), dB	-23.48 (-29.07, -16.56)	-2.19 (-3.55, -0.97)	-27.39 (-30.14, -21.02)	<0.001
PSD, median (IQR), dB	7.70 (2.54, 10.02)	2.14 (1.61, 2.25)	8.21 (5.53, 11.05)	<0.001
Duration of disease, median (IQR), y	1.00 (0.50, 3.00)	0.71 (0.12, 1.00)	1.00 (0.50, 3.00)	0.177
Number of IOP-lowering drugs, median (IQR)	4.00 (3.00, 4.00)	4.50 (2.25, 5.00)	4.00 (3.00, 4.00)	0.527

PACG, and POAG are subtypes of primary glaucoma. Median (IQR) or mean (SD). * Chi-square and Bonferroni adjustment comparisons among two stage groups for variable sex, and Wilcox test for other variables. Abbr.: AACC = acute angle-closure crisis; PACG = primary angle-closure glaucoma; POAG = primary open-angle glaucoma; logMAR = logarithm of minimum angle of resolution; IOP = intraocular pressure; CDR = cup-to-disc ratio; AL = axial length; ACD = anterior chamber depth; ACA = anterior chamber angle; UBM = ultrasound biomicroscopy; RNFL = retinal nerve fiber layer; OCT = optical coherence tomography; MD = mean deviation; PSD = pattern standard deviation.

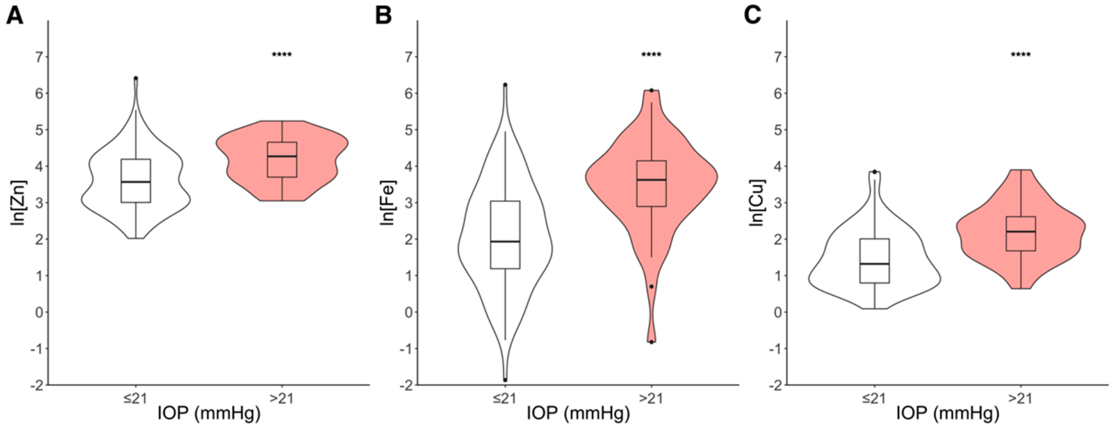

Figure S1. Association between trace metal concentrations in the aqueous humor and IOP levels.

Levels of Zn (A), Fe (B), and Cu (C) are higher in the aqueous humor when IOP > 21 mmHg. The natural logarithmic conversion was performed. Student's t-test. **** $p < 0.001$. Abbr.: IOP = intraocular pressure; Zn = zinc; Fe = iron; Cu = copper.

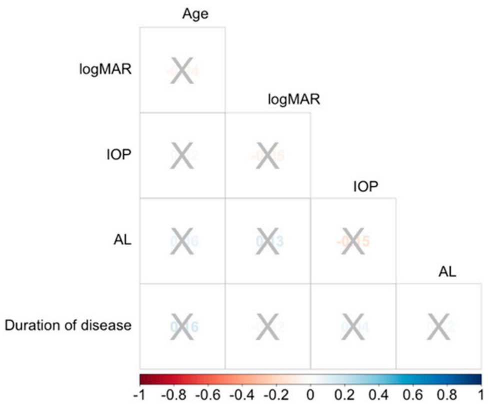

Figure S2.1. Spearman correlation between clinical variables of patients with senile cataract (control).

Spearman correlations. Red and blue indicate negative and positive correlations, respectively. Insignificance is marked with grey crosses. Abbr.: logMAR = logarithm of minimum angle of resolution; IOP = intraocular pressure; AL = axial length.

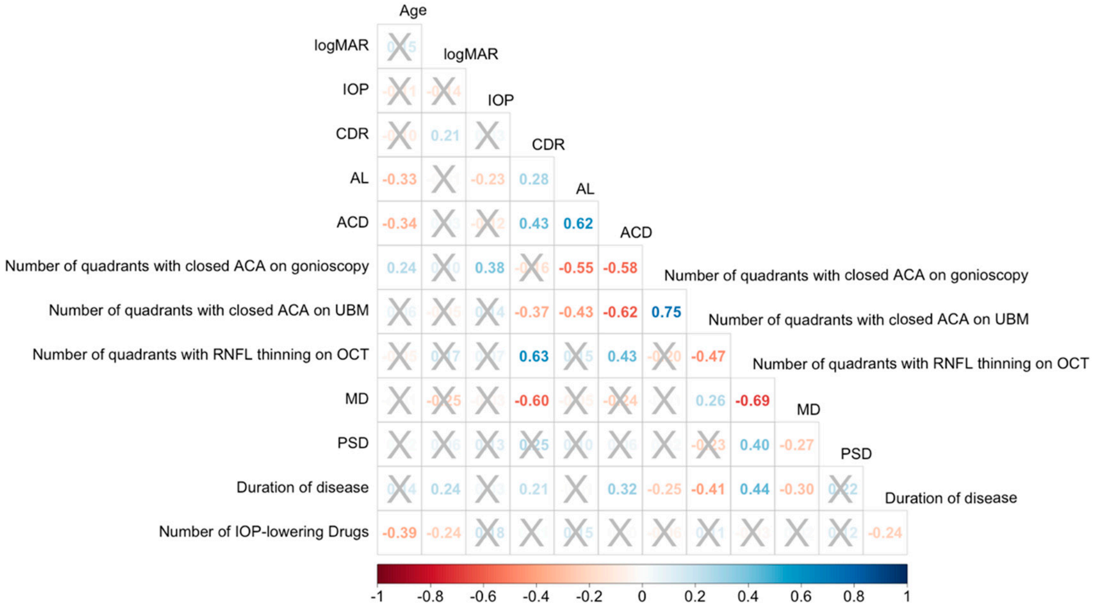

Figure S2.2. Spearman correlation between clinical variables of patients with primary glaucoma.

Spearman correlations. Red and blue indicate negative and positive correlations, respectively. Insignificance is marked with grey crosses. Abbr.: logMAR = logarithm of minimum angle of resolution; IOP = intraocular pressure; CDR = cup-to-disc ratio; AL = axial length; ACD = anterior chamber depth; ACA = anterior chamber angle;

UBM = ultrasound biomicroscopy; RNFL = retinal nerve fiber layer; OCT = optical coherence tomography; MD = mean deviation; PSD = pattern standard deviation.

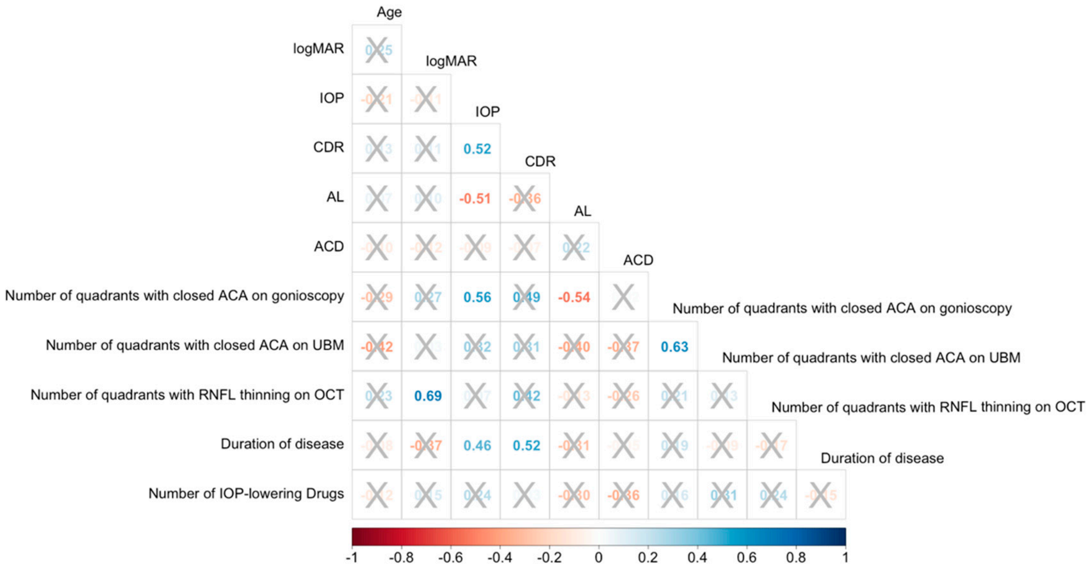

Figure S2.3. Spearman correlation between clinical variables of patients with AACC.

Spearman correlations. Red and blue indicate negative and positive correlations, respectively. Insignificance is marked with grey crosses. Abbr.: AACC = acute angle-closure crisis; logMAR = logarithm of minimum angle of resolution; IOP = intraocular pressure; CDR = cup-to-disc ratio; AL = axial length; ACD = anterior chamber depth; ACA = anterior chamber angle; UBM = ultrasound biomicroscopy; RNFL = retinal nerve fiber layer; OCT = optical coherence tomography.

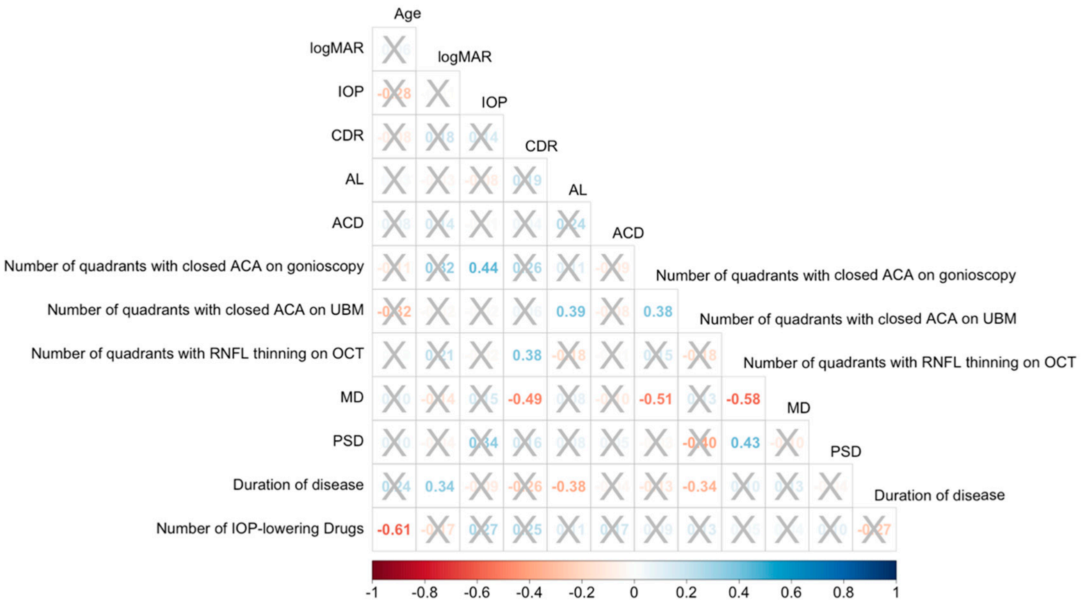

Figure S2.4. Spearman correlation between clinical variables of patients with PACG.

Spearman correlations. Red and blue indicate negative and positive correlations, respectively. Insignificance is marked with grey crosses. Abbr.: PACG = primary angle-closure glaucoma; POAG = primary open-angle glaucoma; logMAR = logarithm of minimum angle of resolution; IOP = intraocular pressure; CDR = cup-to-disc ratio; AL = axial length; ACD = anterior chamber depth; ACA = anterior chamber angle; UBM = ultrasound biomicroscopy; RNFL = retinal nerve fiber layer; OCT = optical coherence tomography; MD = mean deviation; PSD = pattern standard deviation.

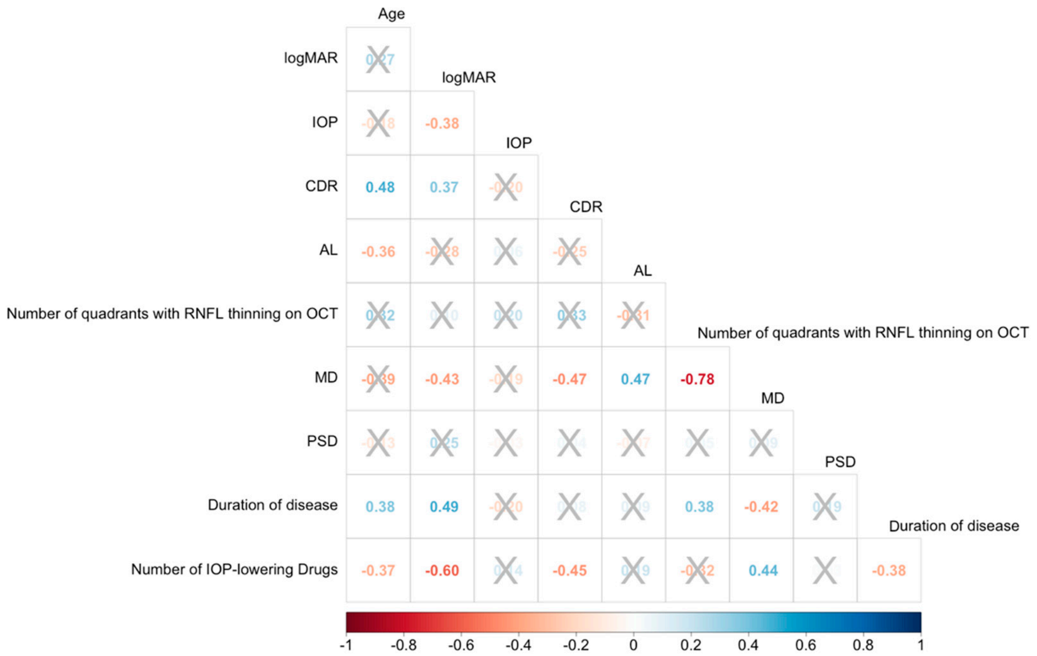

Figure S2.5. Spearman correlation between clinical variables of patients with POAG.

Spearman correlations. Red and blue indicate negative and positive correlations, respectively. Insignificance is marked with grey crosses. Abbr.: POAG = primary open-angle glaucoma; logMAR = logarithm of minimum angle of resolution; IOP = intraocular pressure; CDR = cup-to-disc ratio; AL = axial length; RNFL = retinal nerve fiber layer; OCT = optical coherence tomography; MD = mean deviation; PSD = pattern standard deviation.

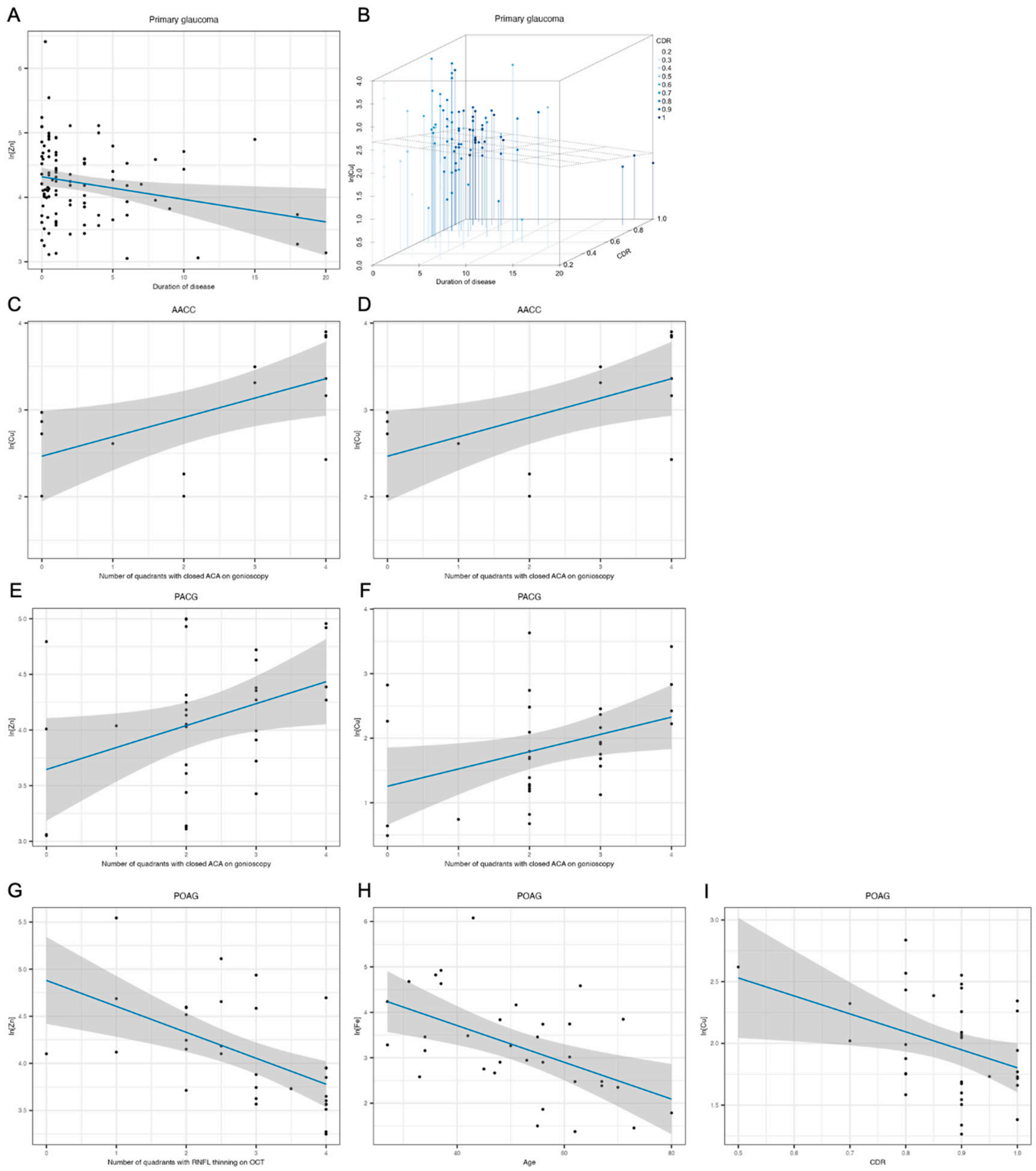

Figure S3. Relationship between trace metals in the aqueous humor and clinical variables in the best regression model.

(A–B) The relationship of duration of disease with ln[Zn], and the relationship of CDR and duration of disease with ln[Cu] in primary glaucoma patients. (C–D) The relationship of number of quadrants with closed ACA on gonioscopy with ln[Fe] and ln[Cu] in AACC patients. (E–F) The relationship of number of quadrants with closed ACA on gonioscopy with ln[Zn] and ln[Cu] in PACG patients. (G–I) The relationship of number of quadrants with RNFL thinning on OCT with ln[Zn], age with ln[Fe], and

CDR with ln[Cu] in POAG patients, respectively. Abbr.: AACC = acute angle-closure crisis; PACG = primary angle-closure glaucoma; POAG = primary open-angle glaucoma; CDR = cup-to-disc ratio; AL = axial length; ACA = anterior chamber angle; RNFL = retinal nerve fiber layer; OCT = optical coherence tomography; Zn = zinc; Fe = iron; Cu = copper.

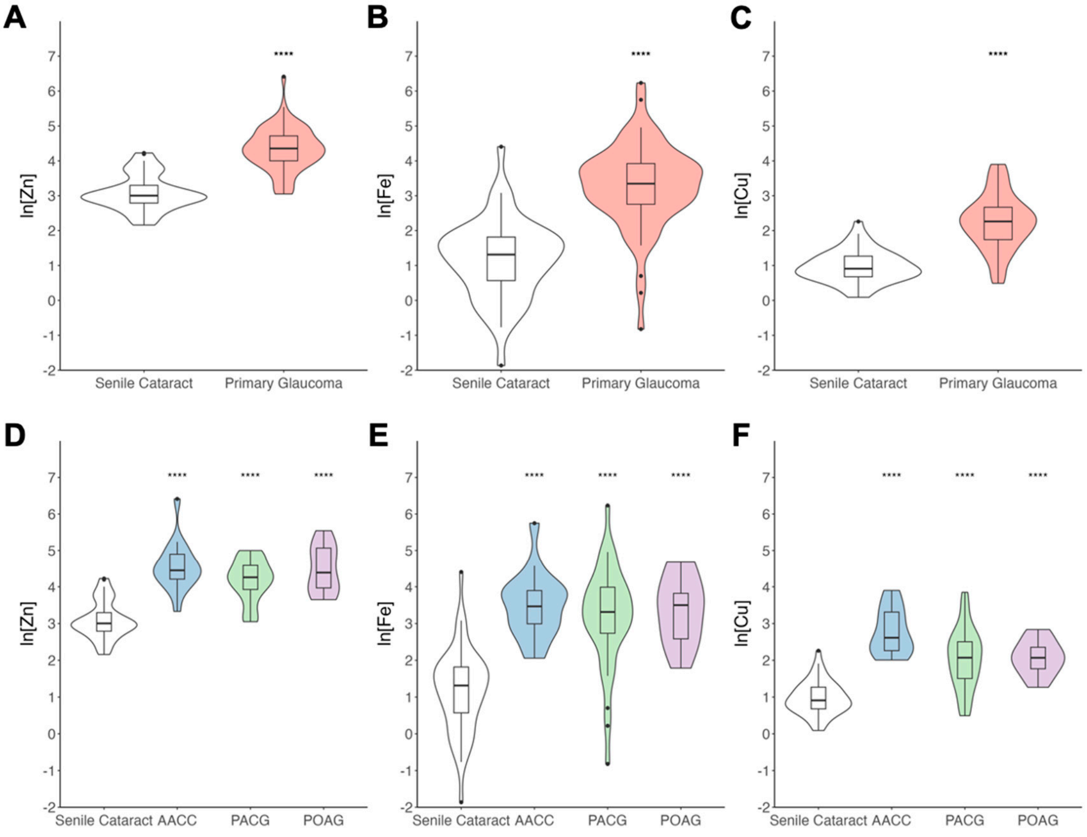

Figure S4. The trace metal concentrations in the aqueous humor of primary glaucoma patients and controls for age- and sex-matched participants.

(A–C) Levels of Zn, Fe, and Cu were elevated in the AH of primary glaucoma patients when compared with those in the AH of controls. The natural logarithmic conversion was performed. Statistical analysis by Student's t-test. **** $p < 0.001$.

(D–F) Zn, Fe, and Cu levels in the AH were elevated in all three subtypes of primary glaucoma patients compared with those in the AH of controls. The AACC group shows greater change in trace metal concentrations than the PACG and POAG groups. The natural logarithmic conversion was performed. Statistical analysis by one-way ANOVA followed by Scheffé post hoc test for Zn and Fe, and Dunnett T3 post hoc

test for Cu. **** p < 0.001 compared with the control group. Abbr.: AACC = acute angle-closure crisis; PACG = primary angle-closure glaucoma; POAG = primary open-angle glaucoma; ln = natural logarithmic transformation; Zn = zinc; Fe = iron; Cu = copper.

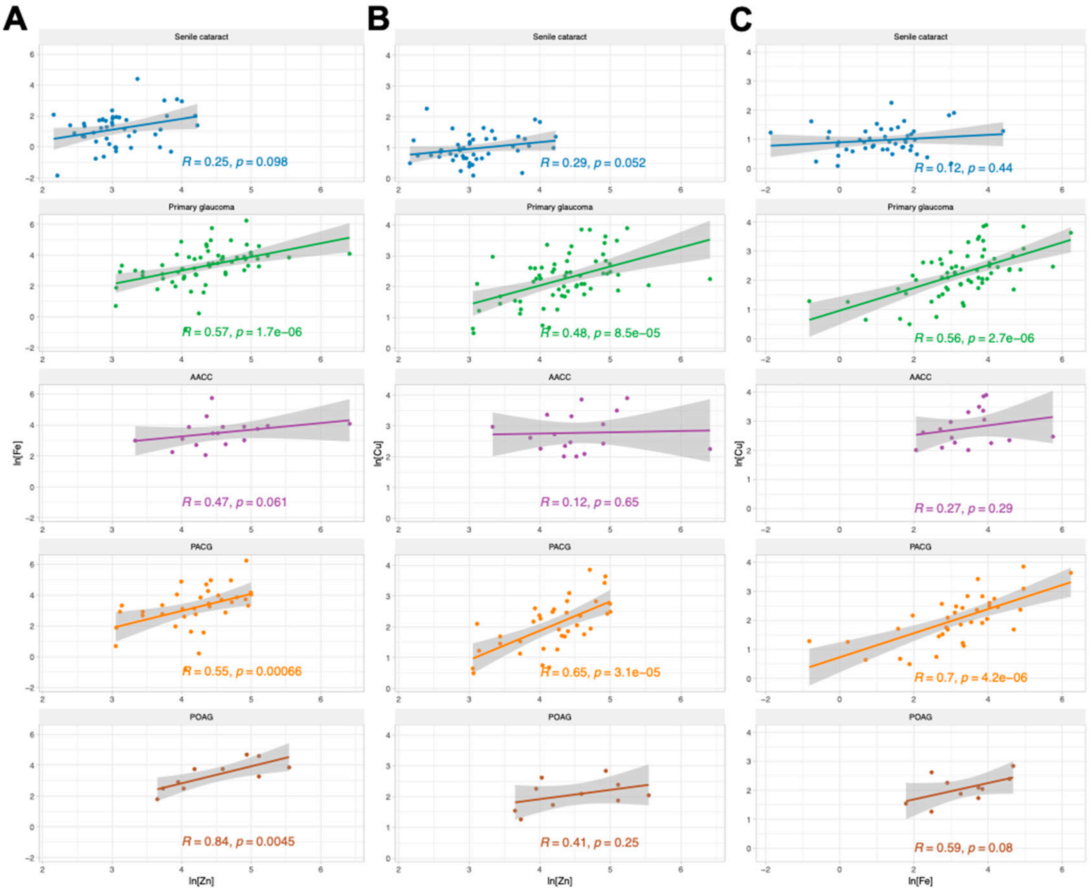

Figure S5. Correlation between trace metal concentrations in the aqueous humor for age- and sex-match participants.

The natural logarithmic conversion of the trace metal concentrations was performed. The Spearman correlations between: (A) ln[Zn] and ln[Fe]; (B) ln[Zn] and ln[Cu]; (C) ln[Cu] and ln[Fe]. Correlation coefficient R and p-values are shown for each group. Abbr.: AACC = acute angle-closure crisis; PACG = primary angle-closure glaucoma; POAG = primary open-angle glaucoma; ln = natural logarithmic transformation; Zn = zinc; Fe = iron; Cu = copper.

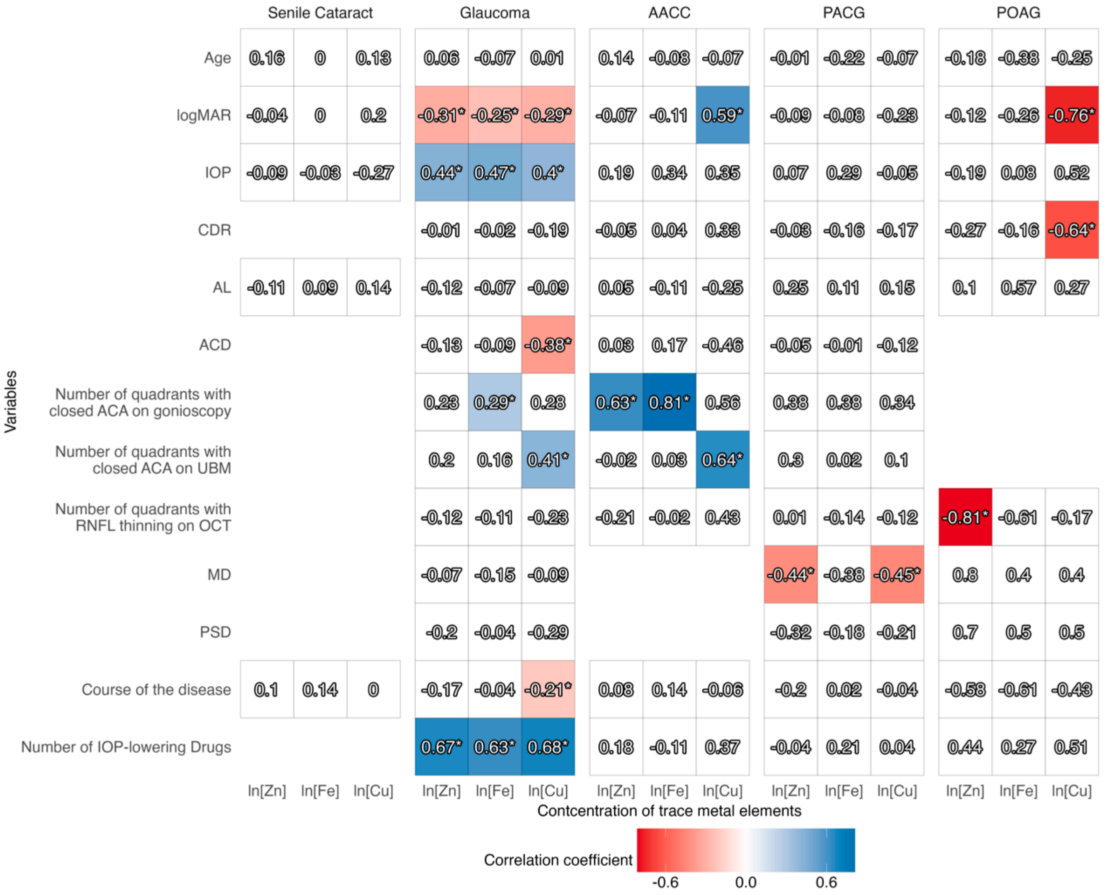

Figure S6. Correlation between trace metal concentrations in the aqueous humor and clinical variables for age- and sex-match participants.

Spearman correlations. Numbers in cells show the correlation coefficient R. Significant correlations ($p < 0.05$) are marked with “*” and cells are colored. Red and blue indicate negative and positive correlations, respectively. Abbr.: AACC = acute angle-closure crisis; PACG = primary angle-closure glaucoma; POAG = primary open-angle glaucoma; logMAR = logarithm of minimum angle of resolution; IOP = intraocular pressure; CDR = cup-to-disc ratio; AL = axial length; ACD = anterior chamber depth; ACA = anterior chamber angle; UBM = ultrasound biomicroscopy; RNFL = retinal nerve fiber layer; OCT = optical coherence tomography; MD = mean deviation; PSD = pattern standard deviation.

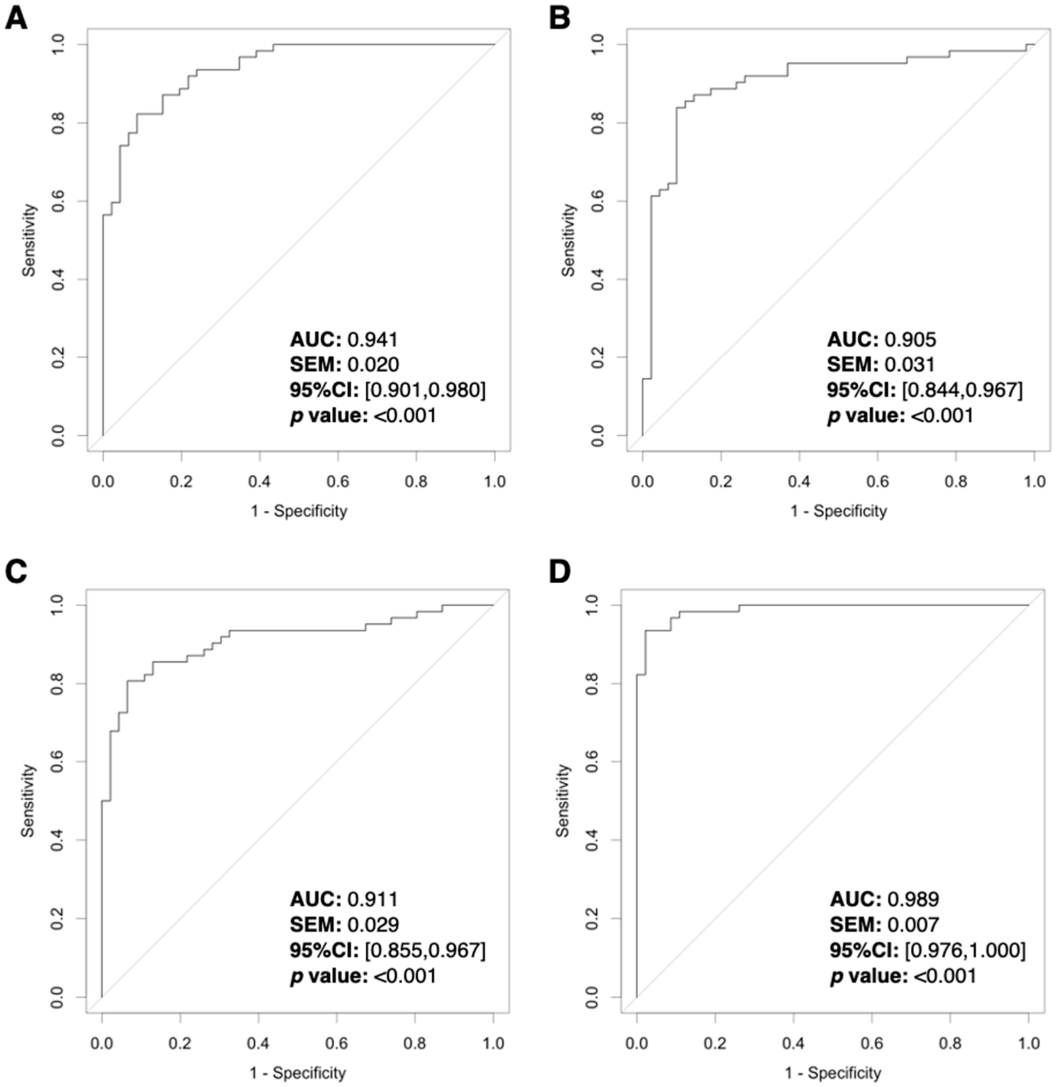

Figure S7. Trace metals as potential biomarkers for discrimination between primary glaucoma and senile cataract for age- and sex-matched participants. The ROC curves of trace metals in the aqueous humor as biomarkers of primary glaucoma. (A) Zn; (B) Fe; (C) Cu; (D) combination of Zn, Fe, Cu, and confounding variables (age, logMAR, and IOP). Abbr.: ROC = receiver operating characteristic; IOP = intraocular pressure; Zn = zinc; Fe = iron; Cu = copper; AUC = area under the ROC curve; SEM = standard error of the mean; CI = confidence interval.

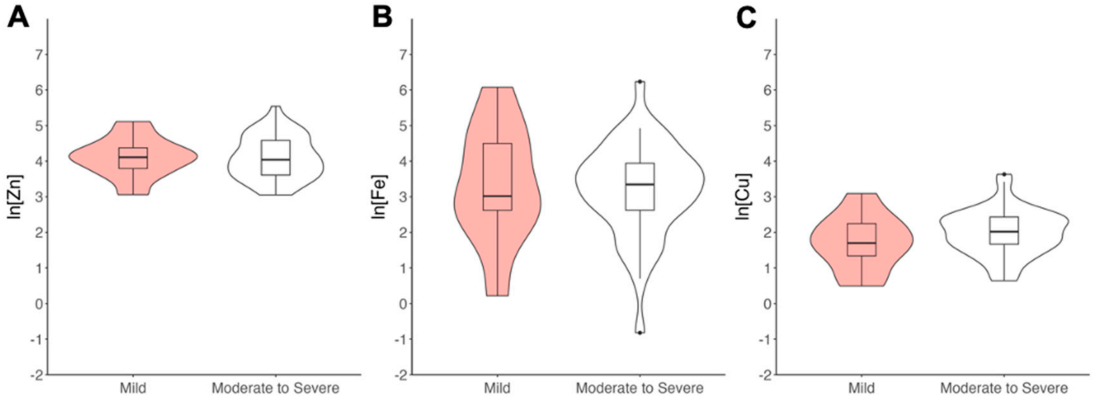

Figure S8. The trace metal concentrations in the aqueous humor by glaucoma stage.

Levels of Zn (A), Fe (B), and Cu (C) don't have a significant difference among mild and moderate to severe stages. Student's t-test. Abbr.: ln = natural logarithmic transformation; Zn = zinc; Fe = iron; Cu = copper.
